# Pharmacological disruption of mSWI/SNF complex activity restricts SARS-CoV-2 infection

**DOI:** 10.1038/s41588-023-01307-z

**Published:** 2023-03-09

**Authors:** Jin Wei, Ajinkya Patil, Clayton K. Collings, Mia Madel Alfajaro, Yu Liang, Wesley L. Cai, Madison S. Strine, Renata B. Filler, Peter C. DeWeirdt, Ruth E. Hanna, Bridget L. Menasche, Arya Ökten, Mario A. Peña-Hernández, Jon Klein, Andrew McNamara, Romel Rosales, Briana L. McGovern, M. Luis Rodriguez, Adolfo García-Sastre, Kris M. White, Yiren Qin, John G. Doench, Qin Yan, Akiko Iwasaki, Thomas P. Zwaka, Jun Qi, Cigall Kadoch, Craig B. Wilen

**Affiliations:** 1 Department of Laboratory Medicine, Yale School of Medicine, New Haven, CT USA; 2 Department of Immunobiology, Yale School of Medicine, New Haven, CT USA; 3 Department of Pediatric Oncology, Dana-Farber Cancer Institute and Harvard Medical School, Boston, MA USA; 4 Broad Institute of MIT and Harvard, Cambridge, MA USA; 5 Program in Virology, Harvard Medical School, Boston, MA USA; 6 Department of Cancer Biology, Dana-Farber Cancer Institute, Boston, MA USA; 7 Department of Medicine, Harvard Medical School, Boston, MA USA; 8 Hillman Cancer Center, University of Pittsburgh Medical Center, Pittsburgh, PA USA; 9 Genetic Perturbation Platform, Broad Institute of MIT and Harvard, Cambridge, MA USA; 10 Department of Microbiology, Icahn School of Medicine at Mount Sinai, New York, NY USA; 11 Global Health Emerging Pathogens Institute, Icahn School of Medicine at Mount Sinai, New York, NY USA; 12 Division of Infectious Diseases, Department of Medicine, Icahn School of Medicine at Mount Sinai, New York, NY USA; 13 Tisch Cancer Institute, Icahn School of Medicine at Mount Sinai, New York, NY USA; 14 Department of Pathology, Molecular and Cell based Medicine, Icahn School of Medicine at Mount Sinai, New York, NY USA; 15 Huffington Center for Cell-based Research in Parkinson’s Disease, Icahn School of Medicine at Mount Sinai, New York, NY USA; 16 Department of Cell, Developmental and Regenerative Biology, Icahn School of Medicine at Mount Sinai, New York, NY USA; 17 Black Family Stem Cell Institute, Icahn School of Medicine at Mount Sinai, New York, NY USA; 18 Department of Pathology, Yale School of Medicine, New Haven, CT USA; 19 Yale Cancer Center, Yale School of Medicine, New Haven, CT USA; 20 Howard Hughes Medical Institute, Chevy Chase, MD USA

**Keywords:** SARS-CoV-2, Epigenetics

## Abstract

Identification of host determinants of coronavirus infection informs mechanisms of viral pathogenesis and can provide new drug targets. Here we demonstrate that mammalian SWItch/Sucrose Non-Fermentable (mSWI/SNF) chromatin remodeling complexes, specifically canonical BRG1/BRM-associated factor (cBAF) complexes, promote severe acute respiratory syndrome coronavirus 2 (SARS-CoV-2) infection and represent host-directed therapeutic targets. The catalytic activity of SMARCA4 is required for mSWI/SNF-driven chromatin accessibility at the *ACE2* locus, *ACE2* expression and virus susceptibility. The transcription factors HNF1A/B interact with and recruit mSWI/SNF complexes to *ACE2* enhancers, which contain high HNF1A motif density. Notably, small-molecule mSWI/SNF ATPase inhibitors or degraders abrogate angiotensin-converting enzyme 2 (ACE2) expression and confer resistance to SARS-CoV-2 variants and a remdesivir-resistant virus in three cell lines and three primary human cell types, including airway epithelial cells, by up to 5 logs. These data highlight the role of mSWI/SNF complex activities in conferring SARS-CoV-2 susceptibility and identify a potential class of broad-acting antivirals to combat emerging coronaviruses and drug-resistant variants.

## Abstract (web summary)

The canonical BRG1/BRM-associated factor (cBAF) complex is recruited by HNF1A/B to angiotensin-converting enzyme 2 (ACE2) enhancers, promoting severe acute respiratory syndrome coronavirus 2 (SARS-CoV-2) infection. Inhibition of the catalytic activity of SMARCA4 precludes *ACE2* expression and reduces susceptibility to SARS-CoV-1 and major SARS-CoV-2 variants.

## Main

While coronavirus disease 2019 vaccines are effective, inadequate vaccination rates, breakthrough infections and viral evolution highlight the need for new antiviral strategies against current and emerging coronaviruses^1^. Enhanced understanding of virus–host interactions at the cellular and molecular levels is critical for the development of both prophylactic and therapeutic approaches^2^. Currently authorized direct-acting antivirals target the viral polymerase (remdesivir and molnupiravir) and viral protease (paxlovid). However, viral resistance, drug–drug interactions and variable efficacy highlight the need for new drug classes with broad activity^3–7^. Host-directed therapeutics provide a particularly promising approach given the potentially higher barrier to drug resistance, increased breadth of activity across coronavirus variants and species and the likelihood of synergy with direct-acting antiviral drugs^8–10^.

Coronavirus entry is mediated by the interaction of the viral spike (S) glycoprotein with a cellular receptor. Three of the seven human coronaviruses including the severe acute respiratory syndrome (SARS)-related beta-coronaviruses (sarbecoviruses) SARS-CoV-1 and SARS-CoV-2, as well as the common cold coronavirus HCoV-NL63, use angiotensin-converting enzyme 2 (ACE2) as a receptor, whereas Middle East respiratory syndrome coronavirus (MERS-CoV) uses dipeptidyl peptidase-4 (DPP4)^11–14^. The S glycoprotein requires proteolytic processing before entry, which can be mediated by several proteases including transmembrane protease serine 2 (TMPRSS2) and the endosomal protease cathepsin L^15–18^. On viral entry, viral RNA is released into the cytoplasm where it is translated and establishes viral replication and transcription complexes before assembling and budding^19–21^.

We recently performed a genome-wide CRISPR–Cas9-based screen to identify host genes essential for highly pathogenic coronavirus infection in African green monkey Vero E6 cells^22^. Many top proviral genes for SARS-CoV-2 encoded subunits of the mammalian SWItch/Sucrose Non-Fermentable (mSWI/SNF) complex: *SMARCA4*, *ARID1A*, *DPF2*, *SMARCE1*, *SMARCB1* and *SMARCC1* (ref. ^22^). These subunit genes have also been identified in other CRISPR screens performed across several different human cell lines^23–25^. mSWI/SNF or BRG1/BRM-associated factor (BAF) complexes are ATP-dependent chromatin remodeling complexes that modulate genomic architecture and gene expression^26–28^. mSWI/SNF complexes are highly conserved across eukaryotes and form three subcomplexes, each with distinct subunit composition, genomic localization properties, nucleosome binding interactions and functions: canonical BAF (cBAF or BAF), polybromo-associated BAF (PBAF) and noncanonical BAF (ncBAF) complexes^29–31^. All mSWI/SNF complexes contain an ATPase subunit, SMARCA4 or SMARCA2 (also known as BRG1 and BRM, respectively), and an array of both shared and complex-specific subunits^29,32^. The cBAF (or BAF) subcomplex is the most stoichiometrically abundant mSWI/SNF complex in mammalian cells, localizing primarily to *cis*-regulatory enhancer elements on the genome^33–38^. As a family, mSWI/SNF complexes represent the most frequently mutated chromatin regulatory entity in human cancer, with >20% of human cancers bearing mutations, including several rare cancers in which mutations are uniformly driving^26,27,36,39,40^. Further, mSWI/SNF genes are frequently perturbed in neurodevelopmental disorders^35,41^. Importantly, well-tolerated and orally bioavailable small-molecule inhibitors and degraders targeting mSWI/SNF family complexes have been recently developed and are currently in human phase I clinical trials across a range of oncology-centered indications (NCT04879017, NCT04891757). However, the mechanism by which mSWI/SNF complexes mediate SARS-CoV-2 infection is unknown.

In this study, we demonstrate that functional mSWI/SNF complexes are required for SARS-CoV-2 infection and viral entry in cell lines and three primary human cell types. We show that mSWI/SNF complex catalytic activity is essential for DNA accessibility at the *ACE2* locus and *ACE2* expression. Enhanced BAF complex targeting to the *ACE2* locus is mediated by the transcription factors HNF1A/B, which bind BAF complexes and direct them to sites of high local HNF1A/B motif density. Finally, inhibition of mSWI/SNF ATP-dependent chromatin remodeling activity using three different SMARCA4/2-specific orally bioavailable small-molecule inhibitors and degraders attenuates *ACE2* expression and reduces infection of numerous SARS-CoV-2 variants. SMARCA4 inhibitors also inhibit infection of a remdesivir-resistant SARS-CoV-2, highlighting the utility for new antiviral drug classes. Notably, mSWI/SNF complexes do not regulate mouse *ACE2* expression, suggesting species-specific regulation of ACE2. Together, these data implicate mSWI/SNF complexes as critical regulators of SARS-CoV-2 infection and new host-directed therapeutic targets.

## Results

### cBAF is required for SARS-CoV-2 infection

**Fig. 1:**
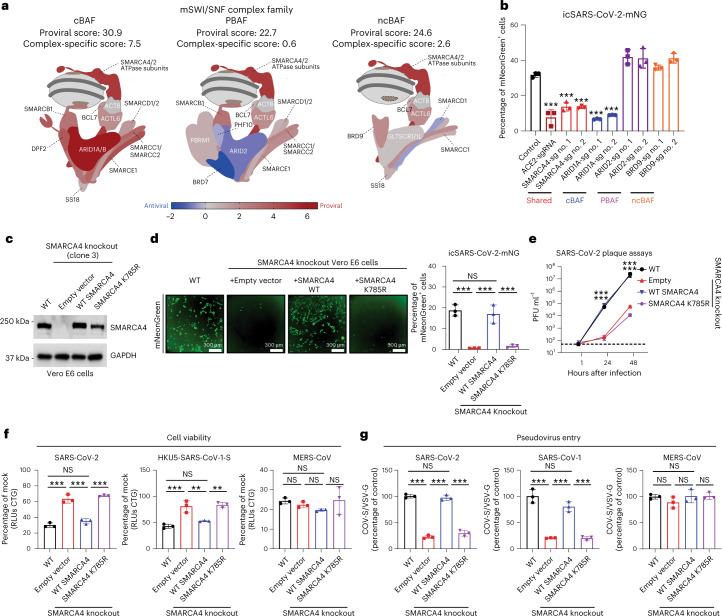
ATPase activity-competent mSWI/SNF complexes are essential for SARS-CoV-2 infection. **a**, Schematic of the three mSWI/SNF family complexes, cBAF, PBAF and ncBAF, with subunits colored according to scores in the Vero E6 SARS-CoV-2 CRISPR–Cas9 screen. The average proviral *z*-scores for each complex are shown. Complex-specific scores represent the sum of two complex-specific subunits, one core subunit and one reader subunit. **b**, Bar graph depicting the percentage of mNeonGreen-expressing Vero E6 cells (control cells or those with polyclonal CRISPR-mediated knockout of shared or unique mSWI/SNF subunits or ACE2) after infection by icSARS-CoV-2-mNG at an MOI of 1. **c**, Immunoblot performed in SMARCA4 knockout Vero E6 cells reconstituted with empty vector, WT SMARCA4 or SMARCA4 ATPase-dead mutant (K785R). **d**, SMARCA4 knockout-complemented and WT Vero E6 cells were infected with icSARS-CoV-2-mNG at an MOI of 1. Infected cells were imaged via fluorescence microscopy (left); mNeonGreen-expressing cell frequency was measured 2 d after infection (right). **e**, SMARCA4 knockout-complemented and WT Vero E6 cells were infected with SARS-CoV-2 at an MOI of 0.1. Virus titer was measured by plaque assay. PFU, plaque-forming unit. **f**, SMARCA4 knockout-complemented and WT Vero E6 cells were infected with SARS-CoV-2 (left), HKU5-SARS-CoV-1-S (middle) and MERS-CoV (right) at an MOI of 0.2. Cell viability relative to a mock-infected control was measured 3 d after infection with CellTiter-Glo (CTG). RLUs, relative light units. **g**, SMARCA4 knockout-complemented and WT Vero E6 cells were infected with VSV pseudovirus (VSVpp): VSVpp-VSV-G; VSVpp-SARS-CoV-2-S (left), VSVpp-SARS-CoV-1-S (middle) and VSVpp-MERS-CoV-S (right). Luciferase relative to the VSVpp-VSV-G control was measured 1 d after infection. Data in **b** and **d**–**g** were analyzed by one-way ANOVA with Tukey’s multiple comparison test. The mean ± s.e.m. are shown. **P < 0.01, ***P < 0.001, NS, not significant. *n* = 3 biological replicates. Data in **c** are representative one of three independent experiments. Source data

**Extended Data Fig. 1:**
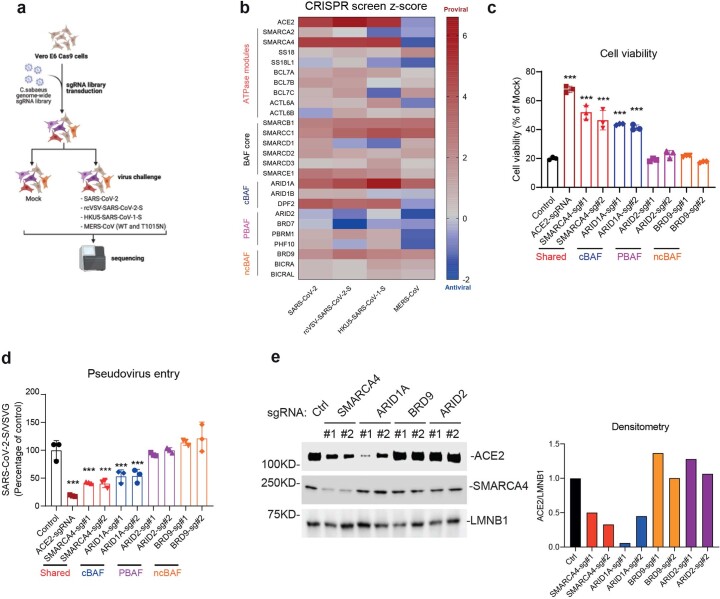
Canonical BAF (cBAF) is essential for SARS-CoV-2 infection at viral entry in Vero E6 cells. **(a)** Schematic of CRISPR/Cas9-based screen to identify SARS-CoV-2 anti- and pro-viral determinants. **(b)** Heatmap of z-scores of mSWI/SNF complex subunits (cBAF, PBAF, ncBAF) in SARS-CoV-2, rcVSV-SARS-CoV-2-S, HKU5-SARS-CoV-1-S, and MERS-CoV CRISPR resistance screens. **(c)** Polyclonal KO of BAF complex subunits and ACE2 KO cells were infected with SARS-CoV-2 at an MOI of 0.2. Cell viability relative to an uninfected control was measured 3 dpi with CellTiter Glo. **(d)** Vero E6 cells were infected with VSV pseudovirus (VSVpp): VSVpp-VSV-G and VSVpp-SARS-CoV-2-S. Luciferase relative to a VSVpp-VSV-G control was measured 1 dpi. **(e)** Left, CRISPR-mediated SMARCA4 knockout was confirmed by Western blot in Vero E6 cells; Right, densitometry analysis of ACE2 levels normalized to LMNB1. Data in (c-d) were analyzed by one-way ANOVA with Tukey’s multiple comparison test. Shown are mean ± SEM. *p < 0.05, **p < 0.01, ***p < 0.001, n = 3 biological replicates. Data in (e) is one representative one of three independent experiments. Source data

Our previous screens identified genes encoding mSWI/SNF complex subunits as critical for SARS-CoV-2 infection, most notably, those subunits corresponding to the cBAF complex^22,29,42^ (Fig. 1a and Extended Data Fig. 1a,b). To determine which of the three mSWI/SNF family complexes regulate SARS-CoV-2 infection, we generated polyclonal knockout cells of SMARCA4 (shared subunit), ARID1A (cBAF-specific subunit), ARID2 (PBAF-specific subunit) and BRD9 (ncBAF-specific subunit) and ACE2 as a positive control in Vero E6 cells using CRISPR–Cas9. We challenged cells with a replication-competent infectious clone of SARS-CoV-2 expressing the fluorescence-based reporter mNeonGreen (SARS-CoV-2-mNeonGreen) and quantified the frequency of infected cells by microscopy^43^. Genetic inactivation of SMARCA4 and ARID1A conferred resistance to SARS-CoV-2-mNG relative to a control single-guide RNA (sgRNA). Inactivation of ARID2 and BRD9 did not reduce infection (Fig. 1b). Consistent with this, disruption of SMARCA4 and ARID1A, but not ARID2 and BRD9, reduced the frequency of SARS-CoV-2-induced cell death (Extended Data Fig. 1c). Similarly, inactivation of SMARCA4 and ARID1A, but not ARID2 or BRD9, inhibited SARS-CoV-2 pseudovirus entry (Extended Data Fig. 1d,e). Collectively, these data demonstrate that the cBAF complex, marked by the complex-specific subunit ARID1A, is required for SARS-CoV-2 entry into cells.

**Extended Data Fig. 2:**
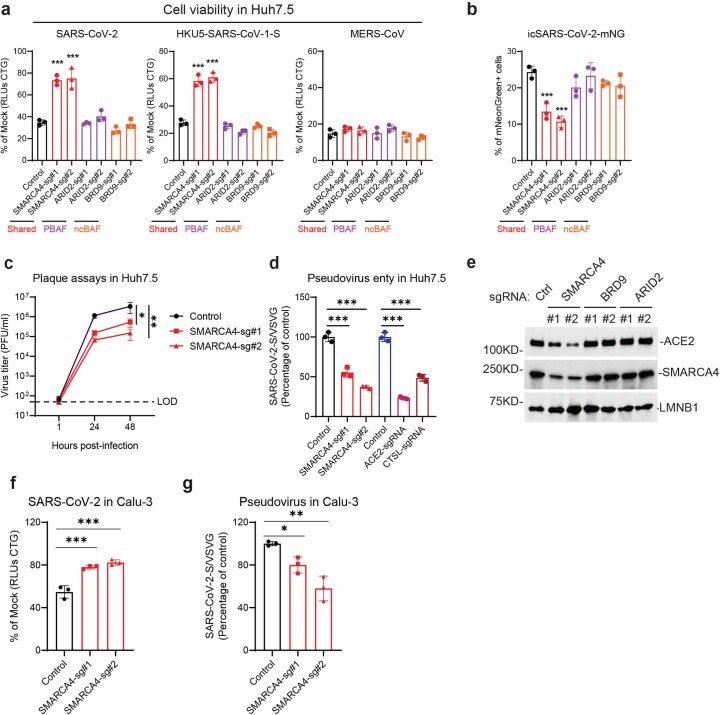
cBAF is essential for SARS-CoV-2 viral entry in human cells. **(a)** BAF complex subunit polyclonal KO pools of Huh7.5 cells were infected with SARS-CoV-2, HKU5-SARS-CoV-1-S and MERS-CoV at an MOI of 0.2. Cell viability relative to an uninfected control was measured 4 dpi using CellTiter Glo. **(b)** Bar graph depicting % mNeonGreen-expressing cells (control cells or those with polyclonal CRISPR-mediated KO of shared or unique mSWI/SNF subunits) following infection by icSARS-CoV-2-mNG at an MOI of 1. **(c)** Huh7.5 cells were infected with SARS-CoV-2 at an MOI of 0.1. Virus titer was measured by plaque assay. Dash line, limit of detection. **(d)** Cells were infected with VSV pseudovirus (VSVpp): VSVpp-VSV-G and VSVpp-SARS-CoV-2-S. Luciferase relative to a VSVpp-VSV-G control was measured 1 dpi. **(e)** CRISPR-mediated SMARCA4 knockout was confirmed by Western blot in Huh7.5 cells. **(f)** Calu-3 cells were infected with SARS-CoV-2 at an MOI of 0.2. Cell viability relative to an uninfected control was measured 3 dpi with CellTiter Glo. **(g)** Calu-3 cells were infected with VSV pseudovirus (VSVpp): VSVpp-VSV-G and VSVpp-SARS-CoV-2-S. Luciferase relative to a VSVpp-VSV-G control was measured 1 dpi. Data in (a-d, f-g) were analyzed by one-way ANOVA with Tukey’s multiple comparison test. Shown are mean ± SEM. *p < 0.05, **p < 0.01, ***p < 0.001. n = 3 biological replicates. Data in (e) is one representative one of three independent experiments. Source data

To assess whether the proviral role of SMARCA4 is restricted to Vero E6 cells, we generated polyclonal SMARCA4 knockout cells in human Huh7.5 and Calu-3 cells, derived from human liver and lung, respectively. Both cell lines endogenously express ACE2 and support SARS-CoV-2 infection. SMARCA4 disruption reduced SARS-CoV-2-induced cell death, SARS-CoV-2 replication and pseudovirus entry in Huh7.5 cells (Extended Data Fig. 2a–e). Similarly, disruption of SMARCA4 in Calu-3 cells conferred protection from SARS-CoV-2-induced cell death and reduced pseudovirus entry (Extended Data Fig. 2f,g). These findings demonstrate a conserved role for ATPase-competent cBAF across cell types and primate species.

### cBAF ATPase activity enables SARS-CoV-2 infection

**Extended Data Fig. 3:**
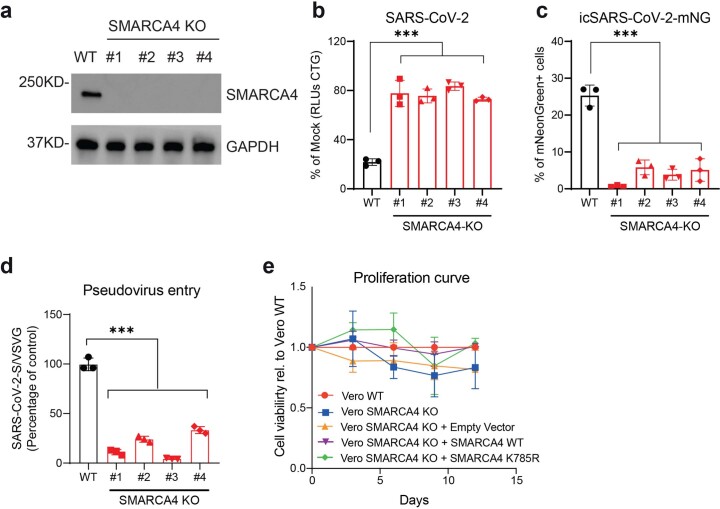
SMARCA4 KO Vero E6 cells are resistant to SARS-CoV-2 infection. **(a)** CRISPR/Cas9-mediated SMARCA4 knockout was confirmed by immunoblot in Vero E6 cells. **(b)** Vero E6 cells were infected with SARS-CoV-2 at an MOI of 0.2. Cell viability relative to an uninfected control was measured 3 dpi with CellTiter Glo. **(c)** Vero E6 cells were infected with icSARS-CoV-2 mNG at an MOI of 1. Infected cell frequency was measured by mNeonGreen expression at 2 dpi. **(d)** SMARCA4 knockout Vero E6 cells were infected with VSV pseudovirus (VSVpp): VSVpp-VSV-G and VSVpp-SARS-CoV-2-S. Luciferase relative to a VSVpp-VSV-G control was measured 1 dpi. **(e)** Cell proliferation of Vero E6 wild type, SMARCA4 KO and KO rescued with empty vector, SMARCA4 wild type or K785R as measured using Cell Titer Glo. Shown are mean ± SEM. n = 3 biological replicates. Data in (a) is one representative one of three independent experiments. Data in (b-d) were analyzed by one-way ANOVA with Tukey’s multiple comparison test. Shown are mean ± SEM. *p < 0.05, **p < 0.01, ***p < 0.001. n = 3 biological replicates. Source data

SMARCA4, the catalytic subunit of mSWI/SNF complexes, most of which associates with cBAF, was second only to ACE2 in our CRISPR screens^22^. We generated four independent single-cell SMARCA4 knockout clones in Vero E6 cells using CRISPR–Cas9 and confirmed knockout efficiency by western blot (Extended Data Fig. 3a). All four SMARCA4 knockout clones were resistant to SARS-CoV-2-induced cell death, SARS-CoV-2 replication and pseudovirus entry (Extended Data Fig. 3b–d). To test whether the observed phenotypes resulted from the absence of SMARCA4 catalytic activity (rather than removal of the five-subunit ATPase module of BAF complexes that SMARCA4 nucleates^29^), we reintroduced wild-type (WT) SMARCA4, an ATPase-dead mutant (K785R) or empty vector control into SMARCA4 knockout cells (clone no. 3) and confirmed expression by western blot (Fig. 1c)^34,44,45^. mSWI/SNF complexes are essential for organism survival and mouse early development; loss of SMARCA4 in mice is embryonic lethal^46–48^. All four SMARCA4 knockout clones exhibited similar proliferation kinetics to WT cells demonstrating that SMARCA4 is not essential for Vero E6 cell replication or viability (Extended Data Fig. 3e). Importantly, reintroduction of WT SMARCA4, but not the K785R mutant, rescued virus replication in knockout cells (Fig. 1d). Similarly, we observed an approximate 3-log reduction of viral replication in SMARCA4 knockout and K785R catalytically inactive cells. This was rescued by complementation with WT SMARCA4 (Fig. 1e).

Next, we asked whether the proviral role of SMARCA4 was specific to SARS-CoV-2. We infected cells with either SARS-CoV-2, a bat coronavirus HKU5 expressing the SARS-CoV-1 S protein (HKU5-SARS-CoV-1-S), or MERS-CoV. The catalytic activity of SMARCA4 was necessary for virus-induced cell death from both SARS-CoV-2 and HKU5-SARS-CoV-1. In contrast, MERS-CoV-induced cell death was independent of SMARCA4 (Fig. 1f). In agreement with these findings, pseudovirus assays indicated that the ATPase activity of SMARCA4 is essential for viral entry of SARS-CoV-2 and SARS-CoV-1 but not MERS-CoV (Fig. 1g). These data demonstrate that SMARCA4 specifically promotes sarbecovirus infection in an ATPase activity-dependent manner.

### cBAF regulates ACE2 levels and is essential for viral entry

**Fig. 2:**
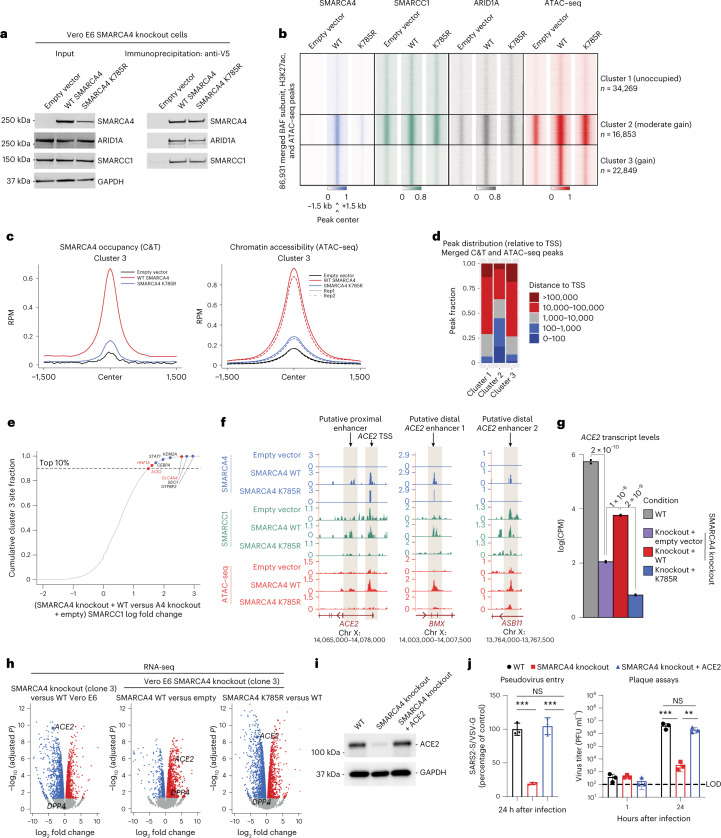
Top-ranked sites of ATPase-active BAF complex occupancy and DNA accessibility include *ACE2*. **a**, Immunoblot performed on nuclear extract (input) and anti-V5 immunoprecipitates from SMARCA4 knockout Vero E6 cells expressing either empty vector, WT V5-SMARCA4 or V5-SMARCA4 K785R. **b**, Heatmap and clustering analysis performed on the merged BAF complex (SMARCA4, SMARCC1 and ARID1A), H3K27ac occupancies (*n* = 1) and ATAC–seq (*n* = 2 biological replicates) peaks performed in Vero E6 cells rescued with the conditions shown in **a**, grouped into three clusters. **c**, Metaplots of SMARCA4 occupancy (C&T) and ATAC–seq peaks at WT SMARCA4-dependent sites (cluster 3). **d**, Distance to TSS distribution of C&T and ATAC–seq merged peaks for all conditions across clusters 1–3 from **b**. **e**, Cumulative distribution function plot reflecting genes nearest to SMARCC1 gained sites in SMARCA4 knockout cells rescued with WT SMARCA4 versus empty vector in cluster 3 from **b**; the top one-tenth fraction reflects genes associated with the top changed sites; sites highlighted in red indicate genes that scored as proviral determinants in the CRISPR screen. **f**, ATAC–seq and C&T tracks at the *ACE2* locus in SMARCA4 knockout Vero E6 cells rescued with empty vector, WT SMARCA4 or SMARCA4 K785R. **g**, Reads per kilobase of transcript per million reads mapped (RPKM) levels for *ACE2* in Vero E6 cells across the conditions shown (*n* = 2 biological replicates). The *P* values shown were calculated in edgeR using a quasi-likelihood negative binomial test. **h**, Volcano plots reflecting gene expression changes (RNA-seq) (*n* = 2 biological replicates) between the conditions shown. **i**, Overexpression of hACE2 in SMARCA4 knockout Vero E6 cells. **j**, VSVpp-based pseudovirus entry assay and plaque assays in WT Vero E6 cells and SMARCA4 knockout cells rescued with human ACE2. Data in **a** and **i** are representative of one of three independent experiments. Data in **j** were analyzed using a one-way ANOVA with Tukey’s multiple comparisons test. The mean ± s.e.m. are shown. **P < 0.01, ***P < 0.001, *n* = 3 biological replicates. The dashed line indicates limit of detection. Source data

**Extended Data Fig. 4:**
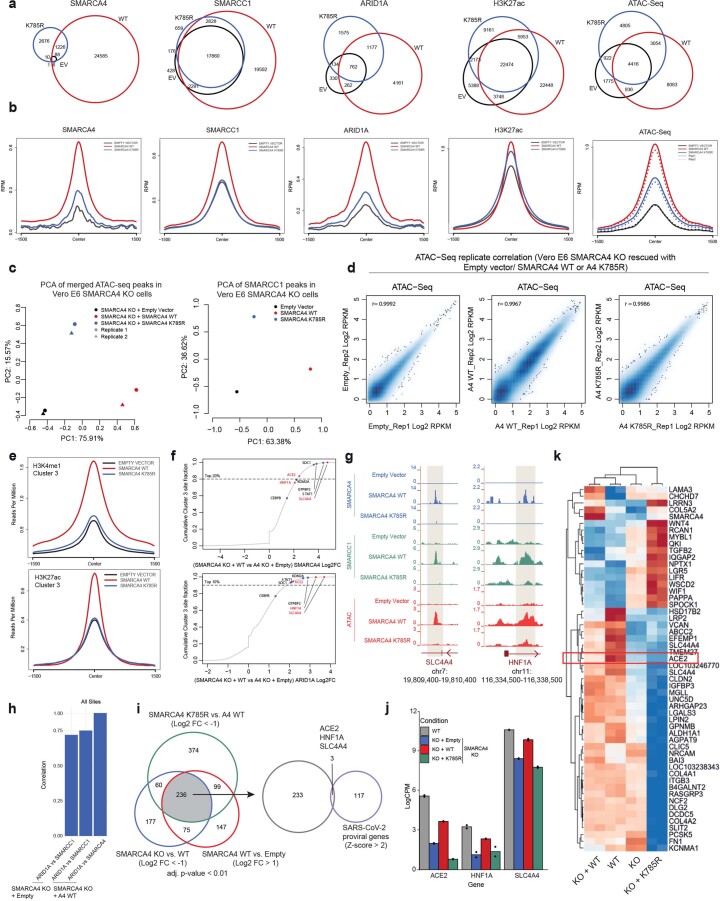
ATPase-competent cBAF promotes ACE2 gene expression. **(a)** Venn diagrams depicting overlap of BAF subunit (SMARCA4, SMARCC1, ARID1A) or H3K27ac histone mark occupancy (CUT&Tag) and ATAC-Seq peaks in rescued SMARCA4 KO Vero E6 cells. **(b)** Metaplots of BAF subunit occupancy (SMARCA4, SMARCC1, ARID1A), H3K27ac histone mark, as measured by CUT&Tag, and ATAC-Seq peaks at all sites in rescued SMARCA4 KO Vero E6 cells. **(c)** PCA plots of merged SMARCC1 occupancy (CUT&Tag) (right) and ATAC-Seq (left) in rescued SMARCA4 KO Vero E6 cells **(d)** Scatter plots of ATAC-Seq replicates in SMARCA4 KO Vero E6 cells expressing Empty control, SMARCA4 WT and SMARCA4 K785R. The correlation coefficient ‘r’ is indicated (top left). **(e)** Metaplots of H3K4me1 and H3K27ac histone mark occupancy (CUT&Tag) at SMARCA4 WT-dependent sites (Cluster 3 from Fig. 2b). **(f)** Cumulative distribution function plots reflecting genes nearest to SMARCA4 and ARID1A gained sites in SMARCA4 KO cells rescued with SMARCA4 WT versus empty vector in Cluster 3 from Fig. 2b; top 1/10th or 1/20th fraction reflect genes associated with top-changed sites, sites highlighted in red indicate genes that scored as pro-viral determinants in CRISPR screen. **(g)** CUT&Tag and ATAC-Seq tracks at *SLC4A4* and *HNF1A* enhancers in rescued SMARCA4 KO Vero E6 cells. **(h)** Correlation between BAF subunit occupancies as profiled by CUT&Tag. **(i)** Venn diagram of overlap between ATPase-competent BAF-dependent transcripts (adj. p-value <0.01, calculated using EdgeR quasi-likelihood negative binomial generalized log-linear model, followed by Benjamini-Hochberg corrections) and proviral genes in the SARS-CoV-2 CRISPR screen (Z-score > 2). **(j)** RPKM levels of ACE2, HNF1A, and SLC4A4 in Vero E6 cells across indicated conditions (n = 2 biological replicates). **(k)** Heatmap of top differentially expressed genes (DEGs) in Vero E6 WT and SMARCA4 KO cells rescued with empty vector, SMARCA4 WT or SMARCA4 K785R. Red rectangle highlights ACE2. Source data

We next sought to define the mechanism underpinning the essentiality of mSWI/SNF, specifically cBAF complexes, in SARS-CoV-2 infection. We performed genome-wide localization studies using CUT&Tag (C&T)^49^ and DNA accessibility profiling using assay for transposase chromatin with sequencing (ATAC–seq)^50,51^ in Vero E6 SMARCA4 knockout cells rescued with empty vector, WT SMARCA4 or the SMARCA4 K785R ATPase-dead mutant (Fig. 2a). Rescue of WT SMARCA4 resulted in increased numbers of BAF complex peaks and ATAC–seq-determined accessible peaks, as expected from previous studies performed in other cellular contexts^34^ (Extended Data Fig. 4a–d). Integration and clustering analyses of these datasets enabled us to define three clusters of genomic sites including: those unoccupied by BAF complexes in all conditions (cluster 1); those with moderate gains in BAF complex occupancy and accessibility on SMARCA4 rescue (cluster 2); and those with substantial gains in BAF targeting and accessibility after expression of only WT SMARCA4 but not the catalytically dead SMARCA4 K785R mutant (ATPase activity-dependent sites) or empty vector control (cluster 3) (Fig. 2b,c). These ATPase activity-dependent sites were largely localized to sites distal to transcription start sites (TSS) and resulted in the establishment of the H3K27ac and H3K4me1 chromatin marks, highlighting their potential role in mediating enhancer accessibility and activation (Fig. 2d and Extended Data Fig. 4e).

We next wanted to identify the most highly increased sites of BAF complex targeting and DNA accessibility within cluster 3 as a strategy to identify gene loci that may underpin the requirement for BAF complexes in SARS-CoV-2 infection. We ranked de novo BAF complex target sites (SMARCC1, SMARCA4 or ARID1A-gained sites) in SMARCA4 knockout cells rescued with WT SMARCA4 versus empty vector within cluster 3 and identified genes closest to these sites. This strategy led us to identify *ACE2* as a top-regulated locus (Fig. 2e). Notably, a number of additional genes localized to SMARCA4-dependent genomic regions were those found to mediate viral infection in our CRISPR screen along with *ACE2*, such as *SLC4A4* and *HNF1A* (Fig. 2e and Extended Data Fig. 4f), thus suggesting that mSWI/SNF complexes regulate a coronavirus susceptibility gene expression axis. We identified three putative enhancer sites, two distal and one proximal, and the promoter of *ACE2*, for which BAF complex occupancy and resulting DNA accessibility depended on the catalytic activity of SMARCA4 (Fig. 2f). Importantly, WT SMARCA4 was required for *ACE2* expression in Vero E6 cells (Fig. 2g,h). Notably, among the 236 transcripts that were dependent on ATPase-competent BAF complexes, three genes, *ACE2*, *SLC4A4* and *HNF1A*, overlapped with screening hits and were dependent on WT SMARCA4 for BAF complex targeting and genomic accessibility (Fig. 2e,h and Extended Data Fig. 4f–i). Taken together, these data underscore the critical role for ATPase-competent BAF complexes in regulating the accessibility of genes critical for SARS-CoV-2 infection, particularly the *ACE2* locus.

Finally, given that *ACE2* was among the most highly regulated genes upon SMARCA4 deletion (Fig. 2h and Extended Data Fig. 4j,k), we sought to determine if SARS-CoV-2 infectivity could be rescued in SMARCA4 knockout cells after rescue with human recombinant ACE2 (hACE2). Indeed, lentiviral expression of hACE2 in SMARCA4 knockout Vero E6 cells rescued SARS-CoV-2 infectivity as assessed by pseudovirus entry and plaque assay (Fig. 2i,j), underscoring that BAF-mediated regulation of ACE2 specifically is responsible for the pro-SARS-CoV-2 phenotype observed.

### Enhanced cBAF complex targeting at ACE2 is mediated by HNF1A

**Fig. 3:**
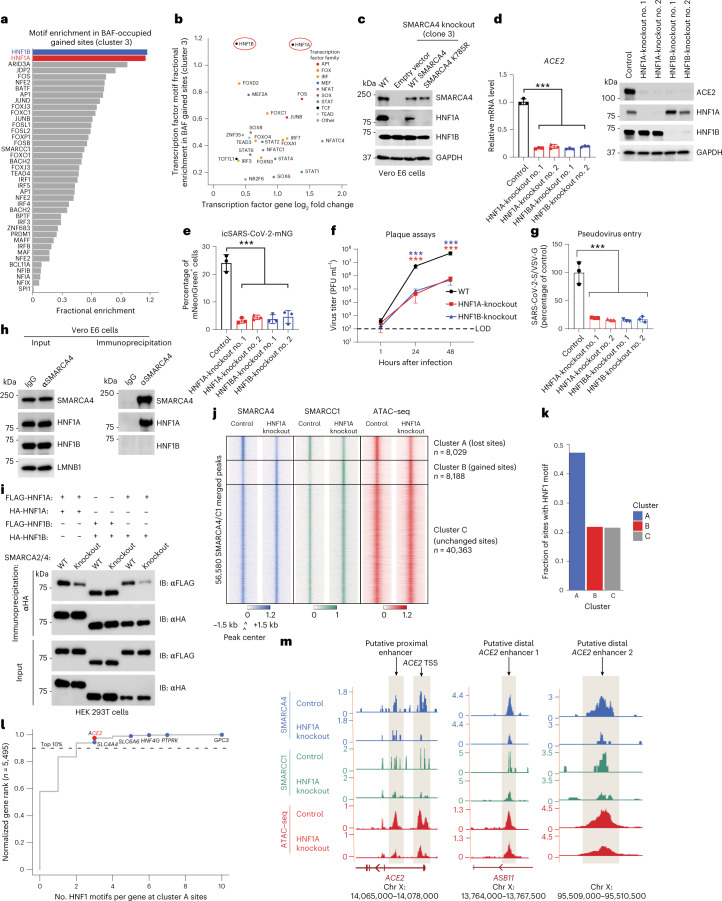
HNF1A–BAF complex binding cooperates with high motif density at the *ACE2* locus to regulate *ACE2* expression. **a**, Transcription factor motif enrichment analysis at BAF-gained sites (cluster 3). **b**, Transcription factor motif enrichment at BAF-occupied gained sites after rescue of Vero E6 SMARCA4 knockout cells with WT SMARCA4 plotted against log_2_ fold change of the transcript levels of the transcription factors (empty vector versus WT SMARCA4 conditions). HNF1A and HNF1B are circled in red. **c**, Immunoblot of HNF1A/B across WT Vero E6 and SMARCA4 knockout cells rescued with empty vector, WT SMARCA4 or SMARCA4 K785R. **d**, *ACE2* expression in HNF1A and HNF1B knockout Vero E6 cells measured by RT–qPCR (left) and immunoblot (right). **e**, WT and HNF1A/B knockout Vero E6 cells were infected with icSARS-CoV-2-mNG at an MOI of 1. The frequency of infected cells was measured using mNeonGreen expression 2 d after infection. **f**, Vero E6 cells were infected with SARS-CoV-2 at an MOI of 0.1. Virus production was measured by plaque assays. **g**, HNF1A and HNF1B knockout Vero E6 cells were infected with SARS-CoV-2 pseudovirus. Luciferase relative to VSVpp-VSV-G control was measured 1 d after infection. **h**, Coimmunoprecipitation of endogenous SMARCA4 and HNF1A in nuclear extracts isolated from Vero E6 cells. **i**, HNF1 dimerization and association studies in WT and SMARCA2/4 double-knockout HEK 293T cells. **j**, Heatmap of SMARCA4 and SMARCC1 merged C&T (*n* = 1) and ATAC–seq (*n* = 2) peaks in control and HNF1A knockout Vero E6 cells, divided into three clusters. **k**, Bar graph depicting the fraction of sites with an HNF1 motif near cluster A (lost sites), cluster B (gained sites) and cluster C (unchanged sites) from **j**. **l**, Normalized gene rank of genes closest to cluster A sites plotted against the number of HNF1 motifs per gene at cluster A sites; selected genes within the top 10% of sites regulated by HNF1A are shown. **m**, C&T and ATAC–seq tracks at the *ACE2* locus in control and HNF1A knockout Vero E6 cells. The data in **c**, **d**, **h** and **i** are representative of one of three independent experiments. The data in **d**–**g** were analyzed using a one-way ANOVA with Tukey’s multiple comparisons test. The mean ± s.e.m. are shown. ***P < 0.001, *n* = 3 biological replicates. Source data

**Extended Data Fig. 5:**
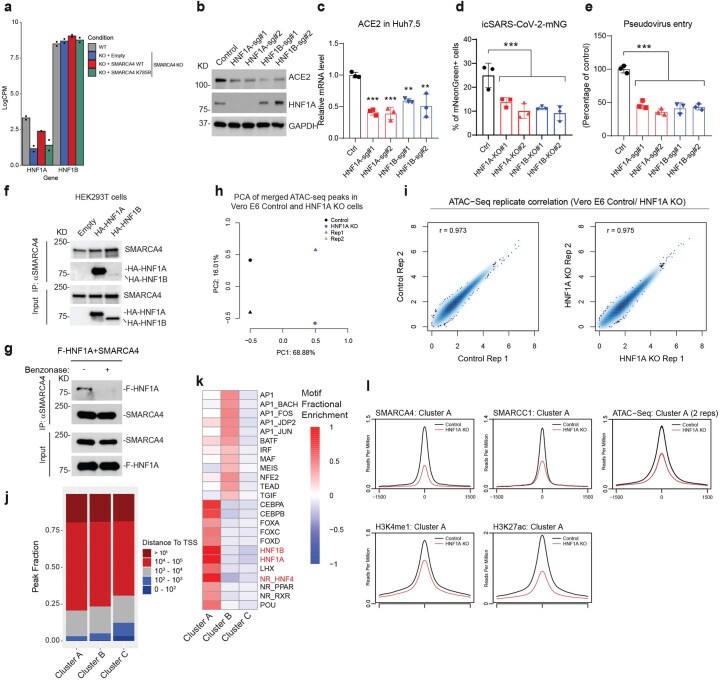
HNF1A directs BAF complex targeting and regulates ACE2 expression. **(a)** RPKM levels for HNF1A and HNF1B in Vero E6 WT and SMARCA4 KO cells rescued with empty vector (Empty), SMARCA4 WT (A4 WT) or SMARCA4 K785R (A4 K785R) (n = 2 biological replicates). **(b-c)** ACE2 expression in HNF1A and HNF1B polyclonal KO Huh7.5 cells as measured by immunoblot (b) and RT-qPCR (c). **(d)** Huh7.5 cells were infected with icSARS-CoV-2-mNG at an MOI of 1. Infected cell frequency was measured by mNeonGreen expression at 2 dpi. **(e)** HNF1A and HNF1B polyclonal KO Huh7.5 cells were infected with VSV pseudovirus (VSVpp): VSVpp-VSV-G, VSVpp-SARS-CoV-2-S. Luciferase relative to the VSVpp-VSV-G control was measured 1 dpi. **(f)** Co-immunoprecipitation of SMARCA4 with HNF1A and HNF1B in HEK293T cells. **(g)** Co-immunoprecipitation of SMARCA4 with Flag-tagged HNF1A (F-HNF1A) in the presence of benzonase in HEK293T cells. **(h)** PCA plot of merged ATAC-Seq peaks in Vero E6 Control and HNF1A KO cells. **(i)** Scatter plots of ATAC-Seq replicates in Vero E6 Control and HNF1A KO cells. The correlation coefficient ‘r’ is indicated (top left). **(j)** Distance to TSS distribution of CUT&Tag and ATAC-Seq merged peaks for all conditions, across Clusters A-C in Vero E6 Control and HNF1A KO cells. **(k)** Heatmap depicting TF motif enrichment in Clusters A-C from Fig. 3j in Vero E6 Control and HNF1A KO cells. **(l)** Metaplots of SMARCA4, SMARCC1, H3K27ac and H3K4me1 chromatin occupancy (CUT&Tag) and DNA accessibility (ATAC-Seq) across Cluster A sites (lost sites) from Fig. 3j in Vero E6 Control and HNF1A KO cells. Data in (b, f-g) is one representative one of three independent experiments. Data in (c-e) were analyzed by one-way ANOVA with Tukey’s multiple comparison test. Shown are mean ± SEM. *p < 0.05, **p < 0.01, ***p < 0.001, n = 3 biological replicates. Source data

We next sought to determine the mechanism by which the *ACE2* locus is regulated by the BAF complex. We performed motif analyses using HOMER v4.9 and MEME on the ATPase activity-dependent sites (cluster 3; Fig. 2b–d) to determine whether specific DNA motifs dominated these genomic regions. Indeed, motifs corresponding to the transcription factors HNF1A and HNF1B were highly enriched (Fig. 3a). Further, HNF1A (but not HNF1B) messenger RNA (mRNA) and protein levels were dependent on SMARCA4 in Vero E6 cells, suggesting that the nuclear abundance of HNF1A and its subsequent targeting to its cognate motifs is controlled by BAF complex activity (Fig. 3b,c and Extended Data Fig. 5a).

Given that the ATPase-dependent sites, including the *HNF1A* gene locus itself, showed the greatest enrichment of HNF1 motifs, we next sought to define the potential role for HNF1A and HNF1B factors in directing BAF complex occupancy and activity to genomic sites such as the *ACE2* locus. To do this, we generated HNF1A and HNF1B knockout Vero E6 cells and confirmed significant reduction of both *ACE2* mRNA and protein levels (Fig. 3d). Notably, genetic depletion of either HNF1A or HNF1B decreased SARS-CoV-2 infection in Vero E6 cells, as measured by mNeonGreen, plaque assays and pseudovirus entry assays (Fig. 3e–g). We confirmed these results in human Huh7.5 cells (Extended Data Fig. 5b–e).

We next asked whether HNF1A, HNF1B or both could bind BAF complexes. To evaluate this, we expressed human influenza hemagglutinin (HA)-tagged HNF1A and HNF1B in HEK 293T cells and performed immunoprecipitation assays. Notably, while HNF1A and HNF1B were expressed at comparable levels, immunoprecipitation of SMARCA4, and hence the BAF complexes, captured HA-HNF1A but not HA-HNF1B. This interaction was dependent on nucleic acids because benzonase treatment abolished binding in the nucleus **(**Extended Data Fig. 5f,g). Consistent with the overexpression data, immunoprecipitation of endogenous BAF complexes in Vero E6 cells also captured only HNF1A but not HNF1B, despite similar nuclear protein levels (Fig. 3h). Given that both HNF1A and HNF1B motifs were enriched in mSWI/SNF-dependent sites and both HNF1 family transcription factors mediated viral infection, yet only HNF1A was shown to bind BAF complexes, we performed reciprocal immunoprecipitations to determine whether HNF1A/B factors dimerized. Consistent with previous studies, we identified homo- and heterodimers of both HNF1A and HNF1B^52–54^. Further, SMARCA4/2 knockout reduced HNF1A homo- and heterodimerization but did not affect HNF1B homodimerization (Fig. 3i), implicating cBAF as a scaffold for HNF1A function. Taken together, these data highlight the specific tethering of HNF1A to BAF complexes, which can either homo- or heterodimerize in a manner dependent on the physical scaffold of the BAF complex ATPase module SMARCA4/2.

We next performed C&T and ATAC–seq to evaluate changes in genome-wide BAF complex targeting and DNA accessibility, respectively, upon HNF1A knockout. Notably, we identified a cluster of 8,029 sites over which BAF complex occupancy and accessibility was substantially reduced after HNF1A knockout (cluster A, lost sites), along with another cluster of gained sites (cluster B, *n* = 8,188) and a large cluster of unchanged sites (cluster C, *n* = 40,363, 71% all sites) (Fig. 3j and Extended Data Fig. 5h–j). Importantly, sites losing BAF complex targeting and associated DNA accessibility upon HNF1A knockout exhibited the highest HNF1A motif density (Fig. 3k and Extended Data Fig. 5k). Further, within cluster A (lost sites), the *ACE2* locus scored among the sites of highest HNF1A motif density (Fig. 3l,m and Extended Data Fig. 5l). Taken together, these results highlight the critical role for the HNF1A–BAF complex binding interaction in directing BAF complex localization and remodeling activities to HNF1A target sites genome-wide. These findings underscore the impact of transcription factor DNA motif density in dictating the degree to which BAF complexes localize and act over a given target sequence.

### SMARCA4 inhibition blocks *ACE2* expression and viral infection

**Fig. 4:**
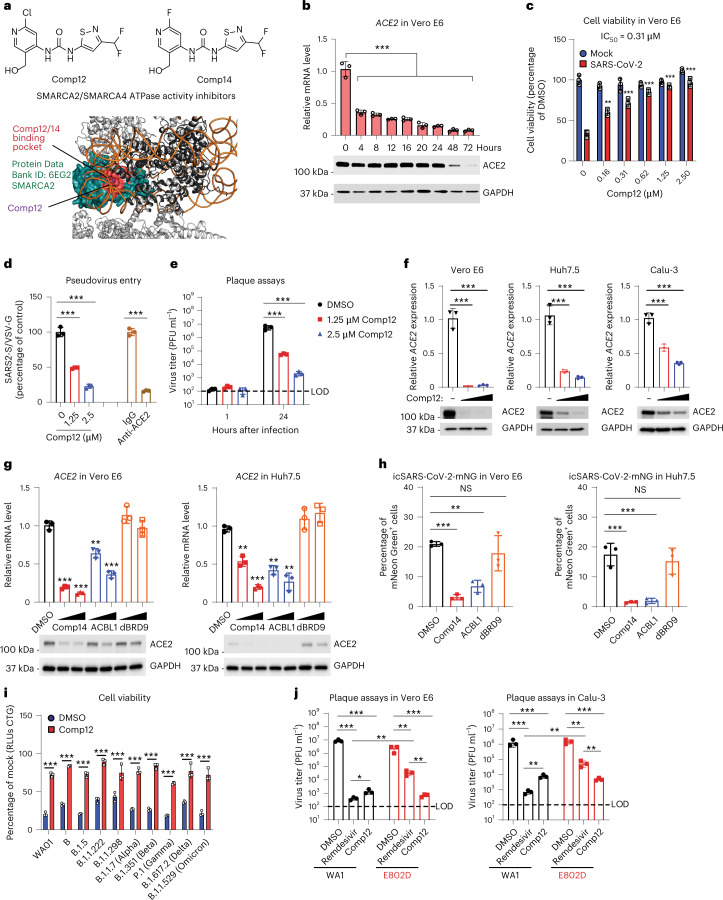
Small-molecule inhibition of the SMARCA4 ATPase of mSWI/SNF complexes downregulates *ACE2* expression and blocks SARS-CoV-2 infection. **a**, Top: chemical structures of the mSWI/SNF SMARCA4/2 ATPase inhibitors, Comp12 and Comp14. Bottom: three-dimensional structure highlighting Comp12 docked in the ATPase site of the SMARCA2/4 ATPase subunit (Protein Data Bank ID: 6EG2). **b**, Vero E6 cells were treated with 1.25 μM Comp12 for the indicated times. ACE2 mRNA and protein levels were measured using RT–qPCR and immunoblot, respectively. **c**, Vero E6 cells were pretreated with Comp12 inhibitors for 2 d and then infected with SARS-CoV-2 at an MOI of 0.2. Cell viability was measured 3 d after infection. **d**, Vero E6 cells were pretreated with 1.25 and 2.5 μM Comp12 for 2 d and then infected with SARS-CoV-2 pseudovirus. The ACE2 antibody was preincubated with cells for 1 h before infection as a positive control. Luciferase relative to VSVpp-VSV-G control was measured 1 d after infection. **e**, SARS-CoV-2 production in Comp12 pretreated Vero E6 cells with the indicated concentrations of Comp12 for 2 d. **f**, *ACE2* transcript and protein levels in Comp12-treated Vero E6, Huh7.5 and Calu-3 cells for 2 d at 1.25 and 2.5 μM. **g**, *ACE2* transcript and protein levels in inhibitor and degrader-treated Vero and Huh7.5 cells for 2 d at 1.25 and 2.5 μM. **h**, Vero E6 and Huh7.5 cells were pretreated with the indicated inhibitors and/or degraders at 2.5 μM for 2 d and then infected with icSARS-CoV-2-mNG. The frequency of infected cells was measured by mNeonGreen expression. **i**, Vero E6 cells were pretreated with 2.5 μΜ Comp12 for 2 d and then infected with the indicated SARS-CoV-2 variants at an MOI of 0.2. Cell viability was measured 3 d after infection. **j**, Vero E6 or Calu-3 cells were pretreated with 2.5 μΜ of Comp12 for 2 d and then infected with the SARS-CoV-2 WA1 and E802D viruses. Virus production was measured by plaque assays 1 d after infection. Data in **b**–**j** were analyzed using a one-way ANOVA with Tukey’s multiple comparisons test. The mean ± s.e.m. are shown. *P < 0.05, **P < 0.01, ***P < 0.001, *n* = 3 biological replicates. Source data

**Extended Data Fig. 6:**
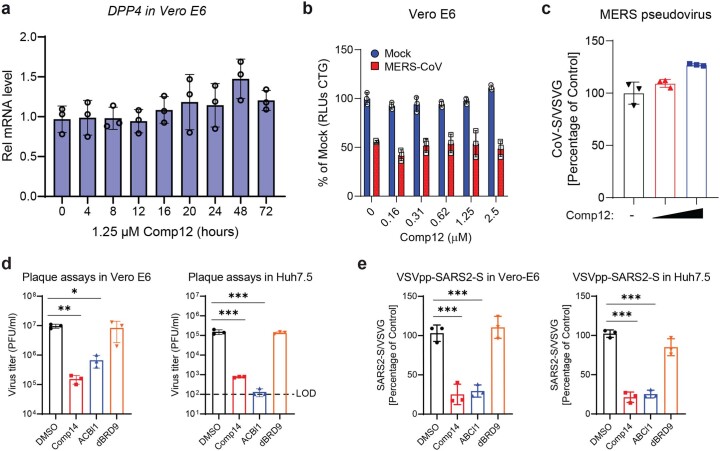
Small molecule targeting of mSWI/SNF complexes inhibits SARS-CoV-2 but not MERS-CoV infection. **(a)** Vero E6 cells were treated with 1.25 μM Comp12 for the indicated times, DPP4 transcript level was measured by RT-qPCR. **(b)** Vero E6 cells were pretreated with Comp12 for 2 days and then infected with MERS-CoV at an MOI of 0.2. Cell viability relative to an uninfected control was measured 3 dpi with CellTiter Glo. **(c)** Vero E6 cells were pretreated with 1.25 or 2.5 μM Comp12 for 2 days and then infected with VSV pseudovirus (VSVpp): VSVpp-VSV-G, and VSVpp-MERS-S. Luciferase relative to a VSVpp-VSV-G control was measured 1 dpi. **(d)** Vero E6 and Huh7.5 cells were pre-treated with indicated inhibitors/degraders (2.5 μM) for 2 days prior to infecting with SARS-CoV-2. Virus replication was measured using plaque assay. Dash line, limit of detection. **(e)** Vero E6 and Huh7.5 cells were pre-treated with indicated inhibitors/degraders (2.5 μM) for 2 days prior to infecting with SARS-CoV-2 pseudovirus to assay for viral entry. Data in (a-e) were analyzed by one-way ANOVA with Tukey’s multiple comparison test. Shown are mean ± SEM. *p < 0.05, **p < 0.01, ***p < 0.001, n = 3 biological replicates. Source data

To investigate the potential of ATPase-competent cBAF complexes as host-directed therapeutic targets for coronavirus disease 2019, we next evaluated the effect of two analogous orally bioavailable small-molecule inhibitors of SMARCA2/4 ATPase activity, Comp12 and Comp14, on SARS-CoV-2 infection^55^ (Fig. 4a). Given the role of mSWI/SNF complex ATPase activity in facilitating DNA accessibility over the *ACE2* locus and *ACE2* gene expression, we evaluated the impact of Comp12 treatment on *ACE2* mRNA and protein expression in Vero E6 cells (Fig. 4b). Notably, mRNA levels of *ACE2* were reduced as early as 4 h after treatment with Comp12, while maximal reduction of protein levels required 48–72 h of treatment (Fig. 4b). Further, Comp12 had no effect on *DPP4* expression, which was similar to the SMARCA4 knockout genetic results (Fig. 2h and Extended Data Fig. 6a). Importantly, we observed dose-dependent inhibition of SARS-CoV-2-induced cell death with Comp12 (half maximal inhibitory concentration (IC_50_) = 310 nM), whereas the cell death induced by MERS-CoV was unaffected (Fig. 4c and Extended Data Fig. 6b). In addition, SMARCA2/4 inhibition with Comp12 reduced SARS-CoV-2 infection and viral entry (Fig. 4d,e) but had no effect on MERS-CoV entry (Extended Data Fig. 6c). We also observed inhibition of *ACE2* expression in Huh7.5 and Calu-3 cells demonstrating that this is generalizable across monkey and human cell lines (Fig. 4f).

Finally, to assess alternate compounds that might block SMARCA4 activity, we tested an analog of Comp12, Comp14 (ref. ^55^) and a completely independent PROTAC degrader of SMARCA2/4 and PBRM1 (ACBI1)^56^. As a negative control, we also tested a PROTAC degrader of the ncBAF-specific protein BRD9 (dBRD9)^57^. Both Comp14 and ACBI1 inhibited *ACE2* expression in both Vero E6 and Huh7.5 cells (Fig. 4g), whereas targeting BRD9 had no effect, which is consistent with our genetic results above. Consistent with ACE2 downregulation, Comp14 and ACBI1 treatment inhibited icSARS-CoV-2-mNG replication, virus production and pseudovirus entry (Fig. 4h and Extended Data Fig. 6d,e).

**Extended Data Fig. 7:**
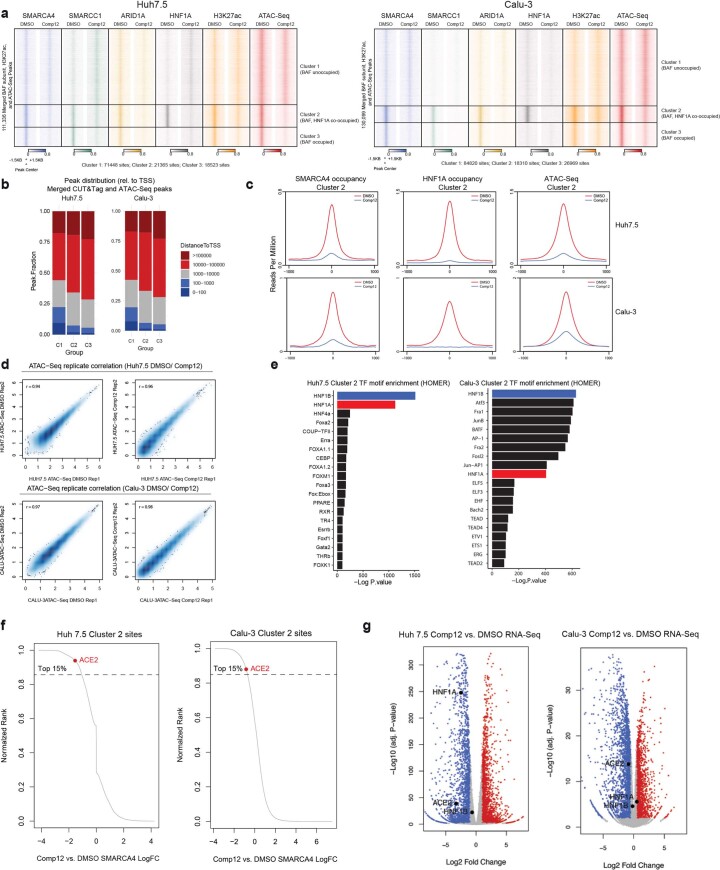
HNF1A and cBAF regulate ACE2 expression in Huh7.5 and Calu-3 cells. **(a)** Heatmap and clustering analyses performed on merged BAF complex (SMARCA4, SMARCC1 and ARID1A), HNF1A, H3K27ac occupancies (n = 1), and ATAC-Seq (n = 2) peaks performed in Huh7.5 and Calu-3 cells treated with DMSO or 1.25 μM Comp12. **(b)** Distance to TSS distribution of CUT&Tag and ATAC-Seq merged peaks for all conditions, across Clusters 1-3 from (a). **(c)** Metaplots of SMARCA4, HNF1A occupancy (CUT&Tag) and ATAC-Seq peaks at BAF, HNF1A co-occupied sites (Cluster 2). **(d)** Scatter plots of ATAC-Seq replicates in DMSO and Comp12 treated cells. The correlation coefficient ‘r’ is indicated (top left). **(e)** Transcription factor motif enrichment analyses at BAF, HNF1A co-occupied sites (Cluster 2) (HOMER uses a binomial distribution to determine enrichment scores and p-values for known motifs). **(f)** Normalized rank of Cluster 2 sites plotted against SMARCA4 occupancy fold change after Comp12 treatment. **(g)** Volcano plots reflecting gene expression changes (RNA-Seq) between indicated conditions.The adj. p-value was calculated using EdgeR quasi-likelihood negative binomial generalized log-linear model, followed by Benjamini-Hochberg corrections.

To confirm that the mechanism of ACE2 downregulation identified in Vero E6 cells was consistent in human cell lines, we profiled BAF complex and HNF1A chromatin occupancy (C&T), DNA accessibility (ATAC–seq) and gene expression (RNA sequencing (RNA-seq)) in Huh7.5 and Calu-3 cells treated with dimethyl sulfoxide (DMSO) or Comp12. In both cases, we identified a cluster of sites co-occupied by HNF1A and cBAF (marked by SMARCA4, SMARCC1 and ARID1A) over which occupancy of both HNF1A and cBAF was lost after treatment with Comp12 (cluster 2) (Extended Data Fig. 7a). These sites also exhibited a corresponding loss in DNA accessibility, were primarily distal in nature and were highly enriched in HNF motifs (Extended Data Fig. 7b–e). Upon ranking cluster 2 sites in both Huh7.5 and Calu-3 cells based on the change in SMARCA4 occupancy in DMSO versus Comp12 conditions, *ACE2* scored again as a top affected locus (Extended Data Fig. 7f). Transcriptional analysis further identified downregulation of *ACE2* transcript levels in Comp12-treated Huh7.5 and Calu-3 cells, underscoring similar regulation of *ACE2* expression by the cBAF complex and HNF1A across the group of cell lines evaluated (Extended Data Fig. 7g).

We next asked whether mSWI/SNF ATPase inhibition could inhibit diverse SARS-CoV-2 variants and virus resistant to a direct-acting antiviral. Like the antiviral effects observed with the prototypic SARS-CoV-2 WA/01, Comp12 protected cells from infection against nine SARS-CoV-2 variants including: B (Germany); B.1.5 (UK); B.1.1.222 (UK); B.1.1.298 (Denmark); B.1.1.7 (Alpha); B.1.351 (Beta); P.1 (Gamma); B.1.617.2 (Delta); and B.1.1.529 (Omicron) (Fig. 4i). We next determined whether Comp12 can overcome drug-resistant virus infection using a remdesivir-resistant SARS-CoV-2 virus, which contained a point mutation, E802D, in the NSP12 RNA-dependent RNA polymerase^3^. Notably, Comp12 exhibited better efficacy against the E802D mutant virus compared to remdesivir **(**Fig. 4j**)**. Taken together, these data indicate that inhibition of mSWI/SNF complex ATPase activity is a viable approach to attenuate diverse sarbecoviruses, including SARS-CoV-2 variants of concern and drug-resistant mutants.

### BAF inhibition blocks SARS-CoV-2 infection in primary cells

**Fig. 5:**
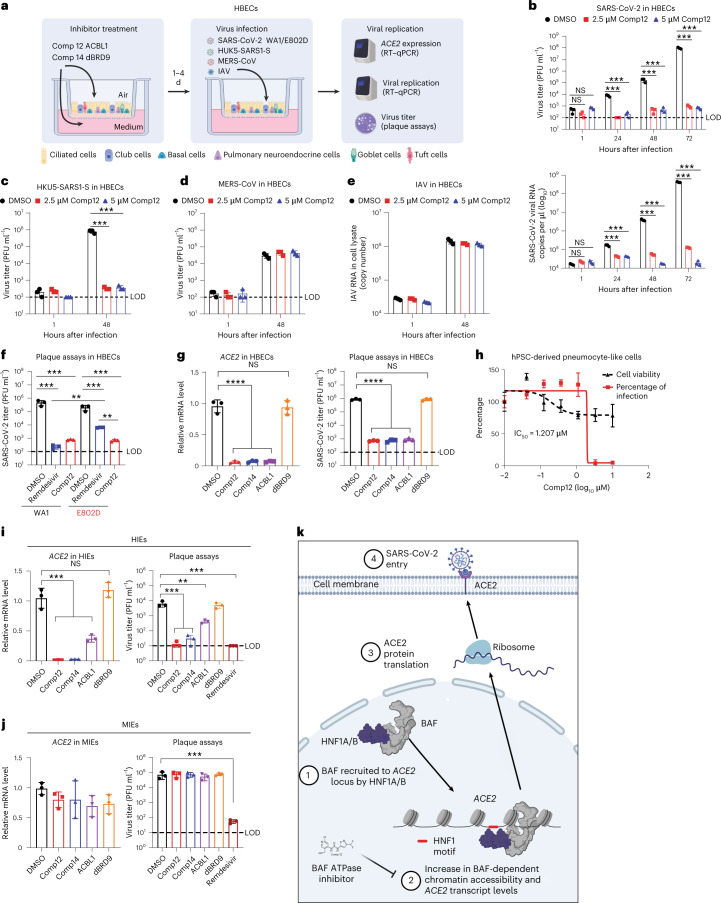
SMARCA4 is required for *ACE2* expression and sarbecovirus susceptibility in primary human cells. **a**, Schematic of SMARCA4/2 ATPase inhibitor treatment and virus infection in primary HBECs. **b**–**e**, HBECs were pretreated with 2.5 and 5 μΜ Comp12 for 4 d and then infected with SARS-CoV-2 (**b**), HKU5-SARS1-S (**c**), MERS-CoV (**d**) and IAV (**e**). Virus replication was measured by plaque assay and/or RT–qPCR. **f**, HBECs were pretreated with 2.5 μΜ Comp12 for 4 d and then infected with SARS-CoV-2 WA1 or E802D virus at an MOI of 0.5. The virus titer was measured using plaque assay. Remdesivir was added right after infection. **g**, *ACE2* expression was measured using RT–qPCR; SARS-CoV-2 replication was measured using a plaque assay after virus infection in HBECs pretreated with 2.5 μΜ of the indicated compounds for 4 d. **h**, hPSC-derived pneumocyte-like cells were pretreated with Comp12 for 2 d and then infected with SARS-CoV-2 at an MOI of 0.1. **i**,**j**, Infectivity was measured by the accumulation of viral nucleoprotein in the nucleus of the cells 2 d after infection. *ACE2* expression and SARS-CoV-2 infection were measured in HIEs (**i**) and MIEs (**j**) pretreated with 2.5 μΜ of the indicated compounds for 3 d except remdesivir, which was added right after virus infection. **k**, Model depicting the mechanism of mSWI/SNF complex-mediated regulation of *ACE2* expression and SARS-CoV-2 entry. In **b**, **c**, **d**, **f**, **g** and **i**, the dashed line indicates the limit of detection. Data in **b**–**j** were analyzed using a one-way ANOVA with Tukey’s multiple comparisons test. The mean ± s.e.m. are shown. **P < 0.01, ***P < 0.001, ****P < 0.0001, *n* = 3 biological replicates. Source data

**Extended Data Fig. 8:**
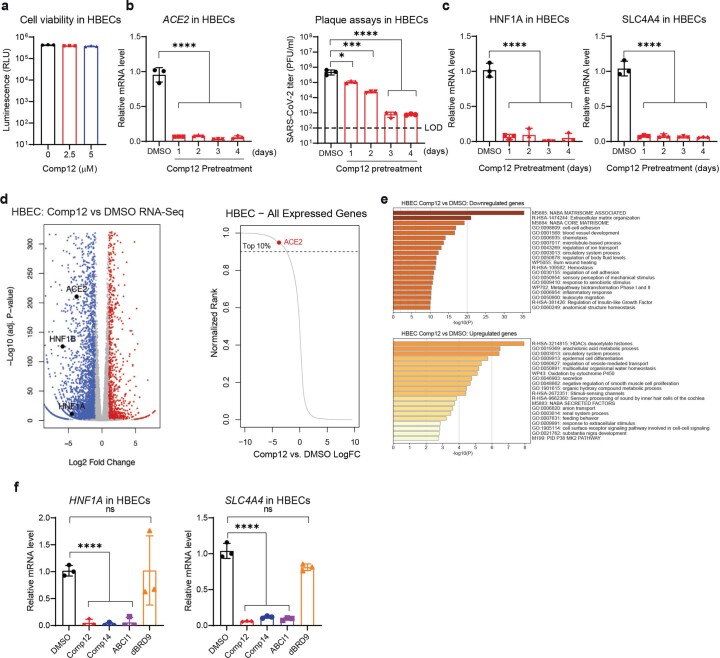
Small molecule targeting of mSWI/SNF complexes in primary cells downregulates ACE2 expression. **(a)** Cell viability of HBECs after 4 days treatment of Comp12 at 2.5 and 5 μM. **(b)** HBECs were pre-treated with Comp12 for the indicated times, ACE2 expression was measured by RT-qPCR. HBECs were pre-treated with Comp12 for the indicated times and then infected with SARS-CoV-2 at an MOI of 0.5. Virus titer in the apical supernatant was measured by plaque assay. Dashed line, limit of detection. **(c)** HBECs were pre-treated with Comp12 at 2.5 μM for the indicated times, HNF1A and SLC4A4 expression was measured by RT-qPCR. **(d)** Volcano plots reflecting gene expression changes (RNA-seq) in HBECs after 4 days treatment of Comp12 at 2.5 μM. **(e)** Pathway analysis (Metascape) in Comp12 treated HBECs (n = 2 RNA-Seq biological replicates). P-values calculated using a hypergeometric test by Metascape. **(f)** HBECs were pretreated with the indicated inhibitors at 2.5 μM for 4 days, HNF1A and SLC4A4 expression were measured by RT-qPCR. Data in (a-c, f) were analyzed by one-way ANOVA with Tukey’s multiple comparison test. Shown are mean ± SEM. *p < 0.05, **p < 0.01, ***p < 0.001, ****p < 0.0001, n = 3 biological replicates. Source data

We next sought to evaluate the antiviral effects of mSWI/SNF complex ATPase inhibition in physiologically relevant primary cells. We first evaluated the impact of SMARCA2/4 ATPase inhibition in primary human bronchial epithelial cells (HBECs) cultured at air–liquid interface (ALI) (Fig. 5a). Remarkably, Comp12 treatment nearly completely blocked SARS-CoV-2 viral replication and virus production in HBECs by approximately 5 logs as measured by plaque assay and quantitative PCR (qPCR) (Fig. 5b). Comp12 did not result in cytotoxicity at the concentrations tested in HBECs (Extended Data Fig. 8a). Consistent with our findings across three cell lines, Comp12 downregulated *ACE2* expression in HBECs (Extended Data Fig. 8b). Comp12 pretreatment of HBECs blocked SARS-CoV-2 infection and downregulated both *ACE2* and *HNF1A* (Fig. 5b and Extended Data Fig. 8b,c). Consistently, ACE2 was one of the most highly downregulated genes in HBECs after Comp12 treatment (Extended Data Fig. 8d). Pathway analysis of differentially regulated genes in response to Comp12 treatment did not identify common antiviral pathways, which is consistent with ACE2 downregulation as the main, if not exclusive, mechanism of action (Extended Data Fig. 8e).

To evaluate the antiviral specificity of Comp12, we challenged Comp12-treated HBECs with HKU5-SARS-CoV-1-S, MERS-CoV and influenza A virus (IAV, A/WSN/1933). As expected, Comp12 blocked HKU5-SARS-1-S replication, which also uses the ACE2 receptor, whereas replication of MERS-CoV and IAV, which use DPP4 and sialic acid as receptors, respectively^13,58,59^, were unaffected (Fig. 5c–e). We next tested the efficacy of Comp12 in overcoming remdesivir-resistant E802D mutant SARS-CoV-2 infection in HBECs. Comp12 treatment restricted E802D virus replication compared to remdesivir treatment, highlighting the potential usefulness of mSWI/SNF inhibition to combat antiviral drug resistance from currently approved drug classes (Fig. 5f). Consistent with Comp12, Comp14 and ACBI1 treatment downregulated *ACE2* and *HNF1A* expression and restricted SARS-CoV-2 infection (Fig. 5g and Extended Data Fig. 8f). Further, Comp12 treatment also restricted SARS-CoV-2 infection in induced pluripotent stem cell-derived ACE2-expressing cells (Fig. 5h). Taken together, these data demonstrate the tolerability, antiviral efficacy and viral specificity of SMARCA2/4 ATPase inhibition in primary human cells.

**Extended Data Fig. 9:**
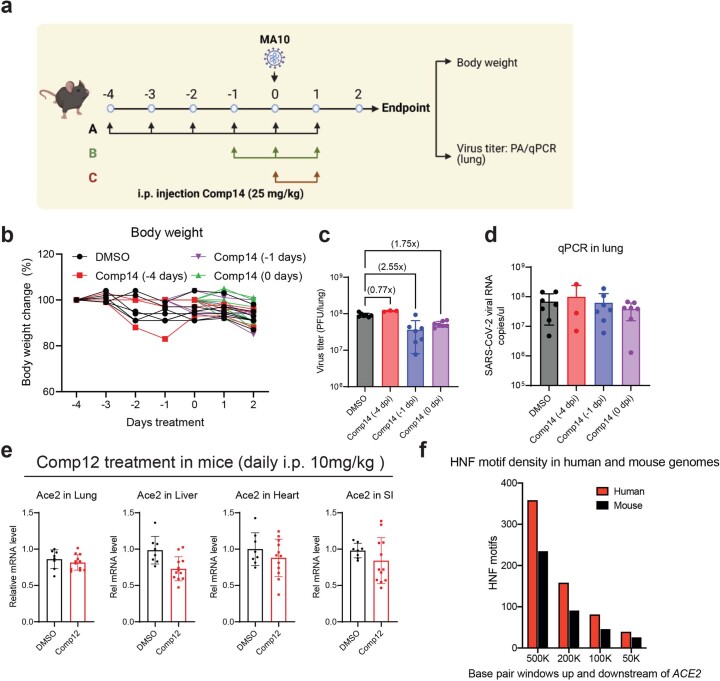
BAF and HNF1A-mediated regulation of ACE2 expression and SARS-CoV-2 infection is not conserved in mice. **(a)** Schematic of Comp14 SMARCA4/2 ATPase inhibitor treatment (i.p. daily at 25 mg/kg) and virus infection in C57BL/6 J mice. **(b)** Body weight change of mice injected with Comp14 following the protocol outlined in (a). **(c**, **d)** SARS-CoV-2 MA10 virus titers (c) and viral genome levels (d) in lung tissues at 2 dpi. **(e)** C57BL/6 J mice were i.p. injected with Comp12 daily at 10 mg/Kg for 5 days and Ace2 expression in different tissues was measured by RT-qPCR. **(f)** HNF1 motif density around the ACE2 locus in human and mouse genomes. Data in (b-d) were analyzed by one-way ANOVA with Tukey’s multiple comparison test. Shown are mean ± SEM. *p < 0.05, **p < 0.01, ***p < 0.001, n = 7 mice, or n = 3 mice. Data in (e) were analyzed by two-tailed t-test, n = 8 mice for DMSO, n = 12 mice for Comp12. Source data

Finally, we investigated the therapeutic potential of cBAF ATPase inhibition in vivo in mice (Extended Data Fig. 9a). Surprisingly, Comp14 treatment of WT C57BL/6J mice did not confer protection from mouse-adapted SARS-CoV-2/MA10 as measured by weight loss and virus titers in the lung (Extended Data Fig. 9b–d). We did not observe significant body weight loss in Comp14-treated mice compared to a DMSO control, demonstrating tolerance of mSWI/SNF inhibition (Extended Data Fig. 9b). Consistent with the lack of antiviral efficacy, *Ace2* expression levels in Comp12-treated mice was comparable to that in DMSO-treated mice (Extended Data Fig. 9e). Given this discrepancy between mouse and cell-based human data, we hypothesized that *Ace2* expression in mice is not regulated by the cBAF-HNF1 axis as in humans. To test this hypothesis, we measured the impact of cBAF ATPase inhibition on *ACE2* expression in human and murine intestinal enteroids (MIEs), which endogenously express high levels of ACE2 (ref. ^60^). Notably, targeting cBAF ATPase activity downregulated *ACE2* expression and blocked SARS-CoV-2 infection in human intestinal enteroids (HIEs) but not in mouse enteroids (Fig. 5i,j), demonstrating fundamental differences between mouse and human ACE2 regulation. Consistent with this, the density of HNF1 transcription factor binding motifs around the *ACE2* locus was markedly reduced in mice compared to humans (Extended Data Fig. 9f). Collectively, these data suggest that the cBAF complex–HNF1 transcription factor axis specifically regulates ACE2 accessibility and expression in primates (Fig. 5k).

## Discussion

In this study, we identified that the transcription factor HNF1A binds to and directs cBAF complexes to the *ACE2* locus. This results in DNA accessibility and induces *ACE2* expression and promotes SARS-CoV-2 susceptibility. This mechanism is conserved across three primate cell lines and three human primary cell types, including airway epithelial cells, and can be therapeutically targeted by orally bioavailable small-molecule inhibitors and degraders of SMARCA4/2 ATPase subunits. mSWI/SNF complex disruption, by either genetic or small-molecule means, potently reduces *ACE2* expression and sarbecovirus infection in cell lines and primary human airway epithelial cells by up to 5 logs, underscoring the potential prophylactic and therapeutic activity of mSWI/SNF inhibitors for current and emerging pandemic sarbecoviruses.

Viral receptor expression is a major determinant of pathogenesis^61–64^. Understanding the molecular mechanisms of the regulation and dynamics of *ACE2* expression may inform host range, tissue and cell type susceptibility, as well as disease severity. Recent studies have revealed that *ACE2* expression increases with age^65^, smoking^66^, infection^67^ and interferons^67–69^; however, the molecular mechanisms governing the dynamic regulation of *ACE2* gene expression are incompletely understood. The detrimental phenotypes associated with *Ace2* germline disruption in mice are attributed to the increase in angiotensin II resulting from *Ace2* deficiency. The effects and tolerability of transient ablation of ACE2, such as that resulting from SMARCA4 inhibition, in adult animals is unclear^70–73^.

Several genome-wide CRISPR–Cas9 screens in the context of SARS-CoV-2 have been now described^22–25,74–77^. SMARCA4 was identified as the second highest hit next to ACE2 in our screen^22^ and was also enriched in a recent screen^23^. SMARCA4 and other mSWI/SNF complex genes were not identified in several other screens, probably owing to the fact that these cell lines ectopically overexpressed ACE2 (that is, in A549 cells and Huh7.5 cells), thus decoupling *ACE2* expression from the endogenous gene regulatory elements needed for mSWI/SNF action, as we show also in this study (Fig. 2i)^24,76,77^. Further, in two screens performed at 37 °C with Huh7.5 cells expressing only endogenous ACE2, ACE2 itself was not detected as a statistically significant hit^25,74^; similarly, in a screen performed at 33 °C with Huh7.5 cells, ACE2 ranked only at 171 out of 19,364 genes^25^. These discrepancies highlight the challenges in detecting genes important for ACE2 regulation in Huh7.5 genome-wide screens and may be due to the limited virus-induced cell death in this cell context. Importantly, we validated the importance of SMARCA4-mediated catalytic activity of mSWI/SNF complexes in Vero E6, Huh7.5, Calu-3 and primary HBEC cells, human induced pluripotent stem cells and HIEs and identified the mechanism by which it regulates *ACE2* expression, reconciling these screening-based discrepancies in the field.

We identified a mechanistic axis by which mSWI/SNF complexes remodel the *ACE2* and *HNF1A* loci, generating DNA accessibility and activating gene expression. HNF1A protein, production of which is dependent on ATPase-active mSWI/SNF complexes, binds mSWI/SNF complexes, guiding complex targeting on the genome to sites of high HNF1A/B motif density, including the *ACE2* locus. Further, our finding that HNF1A but not HNF1B binds BAF complexes, and that homo- and heterodimerization of HNF1 factors requires SMARCA4 and hence fully formed BAF complexes containing the ATPase module^29,34^, suggests a role for BAF complex chromatin binding and nucleosome remodeling for the dimerization of transcription factors such as HNF1A/B. These data will require additional biochemical and structural studies to further define the nature of dimeric transcription factor–BAF complex interactions. Interestingly, HNF1A/B transcription factors exhibit substantial functional differences between mice and humans, with HNF1 transcription factor motifs depleted at the mouse *Ace2* locus relative to the primate *ACE2* locus^78^. Consistent with this, we did not observe *Ace2* downregulation or antiviral activity of SMARCA4 inhibitors in mouse enteroids or in vivo in mice. This highlights a limitation of rodent models for assessing *Ace2* expression and drugs modulating mSWI/SNF complexes or *Ace2*.

New therapeutics are needed for current and emerging coronaviruses to increase antiviral breadth, combat emerging drug resistance and improve tolerability. Currently, host-directed therapeutics predominantly focus on modulating pathogenic immune activation, such as with the steroid dexamethasone^79,80^. In this study, we describe the SMARCA4/2 ATPase, which is specific to mSWI/SNF complexes, as a host-directed therapeutic target independent of immune modulation. SMARCA2/4 ATPase antagonists are currently in phase I clinical trials for SMARCA2/4-dependent cancers, such as uveal melanoma and acute myeloid leukemia (NCT04879017 and NCT04891757) highlighting the feasibility of this approach. Targeting mSWI/SNF complexes in a transient manner offers several potential advantages in the regulation of *ACE2* expression and sarbecovirus infection. First, given the mechanism of mSWI/SNF complexes in regulating genomic accessibility in the host cell, viral antagonism is anticipated to be synergistic with existing, direct-acting antiviral and host immunomodulatory drugs. Second, by downregulating ACE2, SMARCA4 inhibitors can inhibit diverse ACE2-utilizing viruses including HCoV-NL63, SARS-CoV-1, SARS-CoV-2 variants, including remdesivir-resistant forms, and sarbecoviruses recently discovered in bats, which represent a substantial risk for causing future pandemics in humans^81–85^. Taken together, our data suggest that comprehensive studies in humans to evaluate the safety and efficacy of small-molecule antagonists of SMARCA4-mediated mSWI/SNF ATPase activity are warranted and may provide prophylactic and therapeutic benefit for pandemic coronaviruses.

## Methods

### Blinding statement

Data collection and analysis were not performed blind to the conditions of the experiments.

### Data exclusion statement

No animals or data were excluded from the analyses in this study.

### Cells

HEK 293T (cat. no. CRL-3216, ATCC), Vero E6 (cat. no. CRL-1586, ATCC) and Huh7.5 (Washington University in St. Louis) cells were cultured in DMEM with 10% heat-inactivated FCS and 1% penicillin-streptomycin unless otherwise indicated. Calu-3 (cat. no. HTB-55, ATCC) cells were cultured in RPMI 1640 medium with GlutaMAX, 10% FCS, 1% penicillin-streptomycin and 16 ng ml^−1^ of hepatocyte growth factor (Stem Cell Technology) to preserve viability and support robust growth. For Vero E6 and Huh7.5 cells, 5 µg ml^−1^ of puromycin (Gibco) and 5 µg ml^−1^ blasticidin (Gibco) were added as appropriate. For Calu-3 cells, 1 µg ml^−1^ of puromycin was added as appropriate.

### ALI culture of HBECs and infection

Primary HBECs were purchased from Lonza (cat. no. CC-2541) and differentiated in ALI culture as described previously^86^. HBECs were cultured in suspension in PneumaCult-Ex Plus Medium according to the manufacturer’s instructions (STEMCELL Technologies). To generate the ALI cultures, HBECs were plated on collagen-coated transwell inserts with a 0.4-μ pore size (Corning) at 5 × 10^4^ cells per ml per filter and inserted into 24-well culture plates. Cells were maintained for the first 3 d in PneumaCult-Ex Plus Medium then changed to PneumaCult-ALI Medium (STEMCELL Technologies) containing the ROCK inhibitor Y-27632 for 4 d. Fresh medium, 100 μl in the apical chamber and 500 μl in the basal chamber, was provided every day. On day 7, the medium at the apical chambers was removed, while the basal chambers were maintained with 500 μl of PneumaCult-ALI Medium. Medium in the basal chamber was changed every 2–3 d. HBECs were maintained at ALI culture for 28 d, allowing them to differentiate.

Differentiated HBECs were pretreated with inhibitors or DMSO for 1–4 d at both apical and basal sides. HBECs were washed five times with PBS and inoculated with SARS-CoV-2, HKU5-SARS-CoV-1-S, MERS-CoV and IAV from the apical side at a multiplicity of infection (MOI) of 0.5; the cell number per filter support was approximately 5 × 10^5^. After 1 h of incubation at 37 °C, HBECs were rinsed with PBS twice to remove unbound viral particles. Infected HBECs were further maintained under ALI conditions at 37 °C in 5% CO_2_. At different time points, 100 μl of fresh medium was added to the apical surface and the cultures were incubated for 30 min at 37 °C. The supernatants were collected at different times after virus infection and the viruses were titrated by plaque assays on Vero E6 cells. The cell lysates were collected in TRIzol for qPCR analysis.

### HIE and MIE culture and infection

HIEs (J2) derived from a biopsy specimen were kindly provided by M. Estes from Baylor College of Medicine through the Texas Medical Center Digestive Diseases Center Enteroid Core. Protocols for the culture, maintenance and differentiation of HIEs were based on previous studies^87,88^. Briefly, frozen vials of HIEs were thawed out and resuspended in Cultrex Reduced Growth Factor Basement Membrane Extract (BME) (equivalent of Matrigel), Type 2, Select (R&D Systems). The BME mixture (25 µl per well) was plated as droplets onto 24-well tissue culture plates and polymerized at 37 °C for 10 min. Then, 500 µl of growth medium was added to each well and changed every other day. After approximately 7–10 d, HIEs were expanded from 1 well to 3 wells. For HIEs differentiation, growth medium was replaced with an equal volume of differentiation medium and incubated for 4–5 d with medium being changed every other day until use.

MIEs were derived from the ileal tissue of C57BL/6J mice that were approximately 6 weeks old. MIEs were derived by collecting the distal ileal tissue aseptically under the hood. The intestine was flushed and washed with PBS and opened longitudinally to further remove intestinal contents. With sterile scissors, ileal tissues kept on ice in a dish were minced until pieces were small enough to be pipetted with a P1000. Then, 1 ml of collagenase solution (100 mg collagenase type I and 0.001% v/v gentamicin mixed in washing medium) was added to the minced tissue and incubated at 37 °C for 20 min and pipetted every 5 min. The tissue mixture was filtered through a 40-µm strainer and then washed with 9 ml washing medium (DMEM/F12, 100× l-glutamine, 100× penicillin-streptomycin, 10% FCS). Filtrate mixture (containing the crypts) was transferred into a 15-ml falcon tube and were pelleted at 400 *g* for 5 min. Pelleted crypts were suspended into 25 µl BME per well and plated onto 24-well plates. Then, 500 µl of growth medium (50% conditioned medium L-WRN)^88^ was added to each well. After approximately 5 d, MIEs were expanded from 1 well to 3–4 wells. For MIE differentiation, growth medium was replaced with the same volume of differentiation medium and incubated for approximately 4 d with medium changed every other day before use.

Differentiated HIEs and MIE cells were pretreated with the indicated small molecules or DMSO for 3 d in three-dimensional organoid culture. HIEs and MIEs were infected with SARS-CoV-2 at 10^5^ PFU ml^−1^. After 1 h of incubation at 37 °C, the medium was replaced with fresh medium. The cells with medium were frozen and thawed. The supernatants were collected and the viruses were titrated by plaque assays on Vero E6 cells.

### H9 stem cell-derived pneumocyte-like cell differentiation and SARS-CoV-2 infection

Human pluripotent stem cells (hPSC cells) (H9) (obtained from WiCell) were grown with mTeSR (cat. no. 85850, STEMCELL Technologies) on Vitronectin XF-coated (cat. no. 07180, STEMCELL Technologies) tissue culture plates and divided using Gentle Cell Dissociation Reagent every 5–6 d (cat. no. 07174, STEMCELL Technologies). Alveolar differentiation was produced as described previously^89^. On day 9 after differentiation induction, the biosafety level 3 (BSL-3) facility performed viral infections in accordance with Icahn School of Medicine at Mount Sinai (ISMMS)-developed biosafety protocols. Two days before infection, the medium was replaced with 100 µl of medium containing the compound of interest at concentrations 50% greater than those indicated, including a DMSO control. Plates were then transferred into the BSL-3 facility and 4,000 PFU (MOI = 0.1) of SARS-CoV-2/WA1 was added in 50 µl of medium, bringing the final compound concentrations to those indicated. Plates were then incubated for 48 h at 37 °C. After infection, supernatants were removed and cells were fixed with 4% formaldehyde for 24 h before being removed from the BSL-3 facility. Cells were then immunostained for the viral nucleoprotein (an in-house monoclonal antibody 1C7, provided by T. Moran) with a DAPI counterstain. Infected (488 nM) and total cells (DAPI) were quantified using the Celigo (Nexcelom Bioscience) imaging cytometer. Infectivity was measured by the accumulation of viral nucleoprotein in the nucleus of the cells (fluorescence accumulation). Percentage infection was quantified as ((infected cells/total cells) − background) × 100 and the DMSO control was then set to 100% infection for analysis. The IC_50_ and IC_90_ for each experiment were determined using Prism 8 (GraphPad Software). Cytotoxicity was measured using the MTT assay (Roche) according to the manufacturer’s instructions. In uninfected cells, cytotoxicity was measured with same compound dilutions and concurrently with the viral replication assay. All assays were performed in biologically independent triplicates.

### Expression constructs and lentiviral infection

All constructs were PCR-amplified from complementary DNA (cDNA) using Q5 High-Fidelity DNA Polymerase with GC buffer (New England Biolabs). For HA- or FLAG-tag HNF1A and HNF1B, the purified fragments were cloned into a lenti-cytomegalovirus vector containing puromycin resistance. pLX307-WT SMARCA4 and its K785R mutant were as described previously^34^. All constructs were sequence-validated. For lentiviral transduction, cells were transduced at 50% confluency and selected with puromycin 48 h later.

### Viral stocks

To generate viral stocks (Supplementary Table 1) Vero E6 or Vero E6-ACE2-TMPRSS2 cells were inoculated with HKU5-SARS-CoV-1-S (NR-48814), SARS-CoV-2 isolate USA-WA1/2020 (NR-52281), Germany isolate B (NR-52370), B.1.5 (NR-53944), B.1.222 (NR-53945), B.1.1.298 (NR-53953), B.1.1.7 (NR-54000), B.1.351 (NR-54008), P.1 (NR-54982), B.1.617.2 (NR-55611) and MERS-CoV (NR-48813) from BEI resources. B.1.1.529 was isolated from a patient at Yale New Haven Hospital^90^ (icSARS-CoV-2-mNG (provided by the World Reference Center for Emerging Viruses and Arboviruses))^43^ at an MOI of approximately 0.01 for 3 d to generate a P1 stock. The P1 stock was then used to inoculate Vero E6 or Vero E6-ACE2-TMPRSS2 cells for 1–3 d at approximately 50% cytopathic effects. Supernatant was collected and clarified by centrifugation (450 *g* × 5 min) and filtered through a 0.45-µm filter and then aliquoted for storage at −80 °C. Virus titer was determined by plaque assay using Vero E6 cells. All work with infectious virus was performed in a BSL-3 laboratory and approved by the Yale University Biosafety Committee.

### SARS-CoV-2 plaque assays

Vero E6 or Vero E6-ACE2-TMPRSS2 cells were seeded at 4 × 10^5^ cells per well onto 12-well plates. The following day, the medium was removed and replaced with 100 µl of tenfold serial dilutions of virus. Plates were incubated at 37 °C for 1 h with gentle rocking. Subsequently, overlay medium (DMEM, 2% FCS, 0.6% Avicel RC-581) was added to each well. At 2 d after infection, plates were fixed with 10% formaldehyde for 30 min, stained with crystal violet solution (0.5% crystal violet in 20% ethanol) for 30 min and then rinsed with deionized water to visualize plaques.

### SARS-CoV-2 fluorescence-based reporter virus assay

Cells were plated at 2.5 × 10^3^ cells per well onto a 384-well plate and incubated at 37 °C overnight. Cells were infected with icSARS-CoV-2-mNG at an MOI of 1. The frequencies of infected cells were measured by mNeonGreen expression and were assessed 2 d after infection using high-content imaging (Cytation 5, Agilent Technologies). Infection frequencies were calculated as the ratio between mNeonGreen^+^ cells and total cells in bright-field^22^.

### Generation of polyclonal knockout cell lines

Oligonucleotides corresponding to the target sequences were synthesized by Yale Keck Oligo facility (Supplementary Table 2). Double-stranded oligonucleotides were cloned into the lentiCRISPR-V2 vector and cotransfected packaging plasmids into 293T cells. Lentiviral particles were collected and used to transduce Vero E6, Huh7.5 or Calu-3 cells. Infected cells were selected with puromycin for 2 weeks before additional experiments were performed.

To isolate a clonal Vero E6 or Huh7.5 HNF1A and HNF1B knockout cell lines, polyclonal HNF1A and HNF1B knockout cells were diluted and plated onto 96-well plates. Single colonies were grown and clones were screened for HNF1A or HNF1B knockout by western blot.

### Generation of SMARCA4 knockout and complemented cells

Vero E6 SMARCA4 knockout cells were generated by lipofection of Cas9 ribonucleoproteins (RNPs). CRISPR guide RNA (gRNA) were synthesized by Integrated DNA Technologies (Supplementary Table 2). gRNAs were complexed at a 1:1 molar ratio with ATTO550-labeled trans-activating CRISPR RNA (tracrRNA) in TE buffer by heating at 95 °C for 5 min followed by cooling to room temperature to form crRNA–tracrRNA duplexes. Alt-R Cas9 enzyme was combined with the crRNA–tracrRNA duplex at room temperature for 20 min to form RNP complexes in Opti-MEM with 50 µl total volume. Complexes were mixed with Lipofectamine RNAiMAX for 10 min at room temperature before transfection was performed. Single cells were then sorted by flow cytometry and SMARCA4 knockout was confirmed by western blot. SMARCA4 knockout clones were complemented by lentiviral transduction of pLX307 vector or containing full-length SMARCA4 or ATPase-dead mutant K785R with a C-terminal V5 tag. Two days after transduction, puromycin was added and cells were selected for 5 d. The expression of SMARCA4 in complemented cells was detected by western blot.

### SMARCA2/4 inhibitor treatment for cell lines

ACBI1 was purchased from MedChemExpress (cat. no. HY-128359); Comp12 and Comp14 were synthesized as described previously^55^. Vero E6 cells (1.5 × 10^4^) were pretreated with the indicated concentration of Comp12 for 48 h and then infected with SARS-CoV-2 or MERS-CoV at an MOI of 0.2. Cell viability was quantified by CTG 3 d after infection. Vero E6, Huh7.5 and Calu-3 cells (1 × 10^6^) were pretreated with 2.5 µM Comp12 for 48 h, then *ACE2* expression was detected by quantitative PCR with reverse transcription (RT–qPCR) and western blot. Cytotoxicity was not observed in these cell lines during the time and at the concentration of drug used.

### Pseudovirus production

Vesicular stomatitis virus (VSV)-based pseudotype viruses were produced as described previously^22,91^. Briefly, 293T cells were transfected with pCAGGS or pcDNA3.1 vector expressing the CoV S glycoprotein and then inoculated with a replication-deficient VSV virus that contained the expression cassettes for *Renilla* luciferase instead of the VSV-G open reading frame. After an incubation period of 1 h at 37 °C, the inoculum was removed and cells were washed with PBS before medium supplemented with anti-VSV-G clone I4 was added to neutralize residual input virus (no antibody was added to cells expressing VSV-G). Pseudotyped particles were collected 24 h after inoculation, clarified from cellular debris by centrifugation and stored at −80 °C before use.

### Pseudovirus entry assay

A total of 1 × 10^4^ Vero E6, Huh7.5 or Calu-3 cells were seeded in 100 µl total volume in each well of a black-walled clear bottom 96-well plate. The following day pseudovirus was added at a 1:10 final concentration v/v and incubated for 1 d. Cells were lysed with *Renilla* Luciferase Assay System (Promega Corporation) according to the manufacturer’s instructions. Luciferase activity was measured using a microplate reader (Synergy or Cytation 5, BioTek Instruments).

### Small-molecule inhibitor treatment and SARS-CoV-2 (MA10) infection in mice

C57BL/6J mice were injected intraperitoneally daily with Comp14 (25 mg kg^−1^) for 1–4 d or Comp12 (10 mg kg^−1^) for 5 d. For the Comp12 treatment, tissues (lung, liver, heart and small intestine) were collected and homogenized in 1 ml of DMEM supplemented with 2% heat-inactivated FCS and 1% penicillin-streptomycin. Then, 250 μl of homogenate was mixed with 750 μl of TRIzol LS (Invitrogen) and RNA was extracted with Direct-zol RNA MiniPrep Plus Kit (Zymo Research) for *ACE2* expression. For infections, mice were anesthetized with 30% v/v isoflurane diluted in propylene glycol (30% isoflurane) and administered mouse-adapted SARS-CoV-2 MA10 intranasally in 50 μl of PBS. At 2 d after infection, the left lobe of the lung was collected and homogenized in 1 ml of DMEM supplemented with 2% heat-inactivated FCS and 1% penicillin-streptomycin. Lung homogenates were clarified by centrifugation at 3,200 *g* for 10 min and stored in aliquots at −80 °C. Viral burden was measured in lung homogenates by plaque assay on Vero E6 cells. In addition, 250 μl of lung homogenate was mixed with 750 μl of TRIzol LS and RNA was extracted with the Direct-zol RNA MiniPrep Plus Kit according to the manufacturer’s instructions. Mice of both sexes aged between 8 and 10 weeks old were used for this study. All work with SARS-CoV-2 (MA10) was performed in a BSL-3 facility with approval from Environmental Health and Safety and the Institutional Animal Care and Use Committee at Yale University. Mice were randomized based on sex for these experiments.

### RT–qPCR

Total RNA was isolated from cells using the Direct-zol RNA MiniPrep Plus Kit and 1 μg RNA was used for cDNA synthesis. qPCR was carried out using specific primers outlined in Supplementary Table 3.

### Coimmunoprecipitation

Cells were lysed in 1 ml NP-40 lysis buffer (cat. no. J60766, Alfa Aesar). For each immunoprecipitation, 0.5–2 μg of the indicated antibody or control IgG and 30 μl of Protein G Sepharose (GoldBio) were incubated with 0.5 ml lysate for at least 3 h. The Sepharose beads were washed three times with lysis buffer containing 500 mM NaCl. The precipitates were analyzed by western blot.

### Western blot

Cells were collected and lysed in NP-40 lysis buffer. The cell lysates or coimmunoprecipitates were fractionated on SDS–polyacrylamide gel electrophoresis and transferred to a polyvinylidene fluoride membrane. Immunoblotting analyses were performed with the indicated antibodies and visualized either with horseradish peroxidase-coupled goat anti-mouse/rabbit IgG using a chemiluminescence detection system (ChemiDoc MP, BioRad Laboratories) or by IR680/IR800-conjugated anti-rabbit/mouse antibodies (imaged using an Odyssey CLx imaging system, LI-COR Biosciences).

### RNA-seq

Cellular RNA (2 × 10^6^ cells) was extracted in two biological replicates using the Direct-zol RNA MiniPrep Kit. RNA from Vero E6 cells was submitted to the Yale Center for Genome Analysis for library preparation and sequenced on a NovaSeq 6000 instrument. Huh7.5, Calu-3 and HBEC RNA libraries were prepared using the NEBNext Ultra II RNA Library Prep Kit for Illumina (New England Biolabs) and sequenced on a NextSeq 500 instrument.

### ATAC–seq

The omni-ATAC protocol was used to probe DNA accessibility with slight modifications covered below^50^. A total of 100,000 cells per sample were trypsinized and washed with cold PBS to remove trypsin. Cell pellets were lysed in 50 μl cold resuspension buffer (RSB) supplemented with fresh NP-40 (final 0.1% v/v), Tween-20 (final 0.1% v/v), digitonin (final 0.01% v/v) (RSB recipe: 10 mM Tris-HCl, pH 7.4, 10 mM NaCl and 3 mM MgCl_2_). The lysis step was quenched with 1 ml of RSB supplemented with Tween-20 (final 0.1% v/v) and nuclei were pelleted at 500 *g* for 10 min at 4 °C after incubating on ice for 3 min. Nuclei were resuspended in 50 μl transposition reaction mix containing 25 μl 2× Tagmentation DNA Buffer (Illumina), 2.5 μl Tn5 transposase (Illumina), 16.5 μl 1× PBS, 0.5 μl 1% digitonin (final 0.01% v/v), 0.5 μl 10% Tween-20 (final 0.1% v/v) and 5 μl nuclease-free water. The transposition reaction was incubated at 37 °C for 30 min with constant shaking (1,000 r.p.m.) on a thermomixer. Tagmented DNA was purified using the QIAGEN minElute Reaction Cleanup Kit. A standard ATAC–seq amplification protocol with seven cycles of amplification was used to amplify tagmented libraries^92^. Libraries were sequenced on a NextSeq 500 (Illumina) using 37-bp paired-end sequencing.

### C&T

C&T was carried out using a protocol developed by Epicypher (www.epicypher.com/content/documents/protocols/cutana-cut&tag-protocol.pdf) in 8-strip PCR tubes with slight modifications as described below. Briefly, concanavalin A-coated (ConA) magnetic beads were activated with Bead Activation Buffer containing 20 mM HEPES, pH 7.9, 10 mM KCl, 1 mM CaCl_2_, 1 mM MnCl_2_; beads were stored on ice until used. A total of 300,000 cells per sample were trypsinized and pelleted by centrifugation at room temperature (600 *g* for 3 min). Cells were lysed using cold Nuclear Extraction Buffer containing 20 mM HEPES-KOH, pH 7.9, 10 mM KCl, 0.1% Triton X-100, 20% glycerol supplemented with fresh 0.5 mM spermidine and cOmplete, Mini, EDTA-free protease inhibitor (Roche) for 2 min. Nuclei were pelleted by centrifugation (600 *g* for 3 min), resuspended in 100 µl per sample RSB (20 mM HEPES-KOH, pH 7.9, 150 mM NaCl supplemented with fresh 0.5 mM spermidine and cOmplete, Mini, EDTA-free protease inhibitor) and incubated with activated ConA beads at room temperature for 15 min. The nucleus–ConA bead complex was resuspended in Antibody 150 Buffer containing 20 mM HEPES, pH 7.5, 150 mM NaCl, supplemented with fresh 0.5 mM spermidine, protease inhibitor, 0.01% digitonin and 0.5 μg primary antibody per sample. After overnight incubation at 4 °C on a nutator, supernatant was discarded and beads were washed once with Digitonin 150 Buffer containing 20 mM HEPES, pH 7.5, 150 mM NaCl, supplemented with fresh 0.5 mM spermidine, protease inhibitor, and 0.01% digitonin. The ConA–nuclei complexes were then incubated with Digitonin 150 Buffer supplemented with 0.5 μg per sample secondary antibody for 1 h at room temperature on a nutator. They were then washed with Digitonin 150 Buffer twice before resuspension in 50 μl cold Digitonin 300 Buffer containing 20 mM HEPES, pH 7.5, 300 mM NaCl, supplemented with fresh 0.5 mM spermidine, protease inhibitor and 0.01% digitonin. Then, 2.0 μl CUTANA pAG-Tn5 (Epicypher) was added to each sample and incubated on a nutator for 1 h at room temperature. After incubation, beads were washed twice with cold Digitonin 300 Buffer. Targeted chromatin tagmentation and library amplification were carried out according to the Epicypher’s protocol cited above. Libraries were sequenced on a NextSeq 500 instrument using 37-bp paired-end sequencing.

### Antibodies

All primary and secondary antibodies used in this study are listed in Supplementary Table 4.

### Next-generation sequencing data processing

C&T, ATAC–seq and human RNA-seq samples were sequenced with the Illumina NextSeq 500 platform; RNA-seq samples from Vero E6 cells were sequenced with the Illumina NovaSeq 6000 platform. For the RNA-seq data, reads were aligned to either the chlSab2 (NCBI annotation release 100) or the hg19 reference genome using STAR aligner v2.7.3a^93^ with the parameters --winAnchorMultimapNmax 200--outFilterMultimapNmax 100--quantMode GeneCounts. bigWig files were generated using the deepTools v.3.1.3 bamCoverage function^94^ with the normalizeUsingRPKM option. Output gene count tables from STAR were used as input into the edgeR v.3.12.1 R software package^95^ to evaluate differential gene expression. For the ATAC–seq data, read trimming was carried out using Trimmomatic v.0.36 (ref. ^96^) followed by alignment, duplicate read removal and read quality filtering using Bowtie2 (ref. ^97^), Picard v.2.8.0 (http://broadinstitute.github.io/picard/) and SAMtools v.0.1.19 (ref. ^98^), respectively. ATAC–seq peaks were called with MACS2 v.2.1 (ref. ^99^) using the BAMPE option and a broad peak cutoff of 0.001. For ATAC–seq track generation, output BAM files were converted into bigWig files using the MACS2 and UCSC utilities^100^ to display coverage throughout the genome in reads per million (RPM) values. For the C&T libraries, the CutRunTools pipeline was leveraged to perform read trimming, quality filtering, alignment, peak calling and track building using default parameters^101^. All sequencing data analyzed in this study have been deposited at the NCBI’s Gene Expression Omnibus (GEO) under accession no. GSE186201.

### C&T and ATAC–seq data analyses

Heatmaps and metaplots displaying signals aligned to peak centers were generated using ngsplot v.2.63 (ref. ^102^). RPM values were quantile-normalized across samples and *k*-means clustering was applied to partition the data into groups. The bedtools multiIntersectBed and merge functions were used for peak merging^103^; distance-to-TSS peak distributions were computed using Ensembl gene coordinates provided by the UCSC genome browser. Principle component analysis was performed using the wt.scale and fast.svd functions from the corpcor R package on C&T quantile-normalized log_2_-transformed RPKM values within merged peaks^104,105^. Transcription factor motif positions within peaks were identified using the MEME FIMO v.4.12.0 software^106^ with position frequency matrices curated previously^107^; motif fractions of occurrence within clusters of peaks were computed using in-house scripts.

### Quantification and statistical analysis

All statistical analyses were performed in Prism 8 unless otherwise stated. The error bars represent the s.e.m. Viral shedding over time was analyzed by repeated-measures analysis of variance (ANOVA). All statistically analyzed pairwise comparisons are indicated with a bar and the *P* value is represented by *P < 0.05, **P < 0.01, ***P < 0.0001 and ****P < 0.00001. The absence of a bar indicates that no statistical pairwise comparisons were made. *P* values are listed in Supplementary Table 5.

### Ethics statement

This research complies with all relevant ethical regulations and the research protocols are approved by the Yale School of Medicine, Dana-Farber Cancer Institute and ISMMS. All animal work was approved by the Institutional Animal Care and Use Committee at Yale University School of Medicine according to its guidelines. All infection work was performed in an animal BSL-3 facility at Yale University School of Medicine.

### Reporting summary

Further information on research design is available in the Nature Portfolio Reporting Summary linked to this article.

## Online content

Any methods, additional references, Nature Portfolio reporting summaries, source data, extended data, supplementary information, acknowledgements, peer review information; details of author contributions and competing interests; and statements of data and code availability are available at 10.1038/s41588-023-01307-z.

## Supplementary information

Supplementary Information Supplementary Tables 1–5.

Reporting Summary

## Data Availability

The RNA-seq, CUT&Tag and ATAC–seq data generated during this study are available at GEO under accession no. GSE186201. Source data are provided with this paper.

## Source data

Source Data Fig. 1 Statistical source data.
Source Data Fig. 2 Statistical source data.
Source Data Fig. 3 Statistical source data.
Source Data Fig. 4 Statistical source data.
Source Data Fig. 5 Statistical source data.
Source Data Extended Data Fig. 1 Statistical source data.
Source Data Extended Data Fig. 2 Statistical source data.
Source Data Extended Data Fig. 3 Statistical source data.
Source Data Extended Data Fig. 4 Statistical source data.
Source Data Extended Data Fig. 5 Statistical source data.
Source Data Extended Data Fig. 6 Statistical source data.
Source Data Extended Data Fig. 8 Statistical source data.
Source Data Extended Data Fig. 9 Statistical source data.
Source Data Fig. 1 Unprocessed western blots.
Source Data Fig. 2 Unprocessed western blots.
Source Data Fig. 3 Unprocessed western blots.
Source Data Fig. 4 Unprocessed western blots.
Source Data Extended Data Fig. 1 Unprocessed western blots.
Source Data Extended Data Fig. 2 Unprocessed western blots.
Source Data Extended Data Fig. 3 Unprocessed western blots.
Source Data Extended Data Fig. 5 Unprocessed western blots.

## Code availability

No custom code was generated for this study.
