# Supplementary material for: Pharmacological disruption of mSWI/SNF complex activity restricts SARS-CoV-2 infection
Source: Nat Genet. 2023 Mar 9;55(3):471–83. doi: 10.1038/s41588-023-01307-z (PMC10011139; doi:10.1038/s41588-023-01307-z)
Supplement: Supplementary file 1 — Supplementary Tables 1–5. [file 41588_2023_1307_MOESM1_ESM.pdf]

nature genetics

Article

https://doi.org/10.1038/s41588-023-01307-z

Pharmacological disruption of mSWI/SNF complex activity restricts SARS-CoV-2 infection

In the format provided by the authors and unedited

Supplementary Table 1

VIRUS	SOURCE	IDENTIFIER
SARS-CoV-2, isolate US-WA1/2020	BEI Resources	NR-52281
SARS-CoV-2, Germany isolate B	BEI Resources	NR-52370
SARS-CoV-2, B.1.5	BEI Resources	NR-53944
SARS-CoV-2, B.1.222	BEI Resources	NR-53945
SARS-CoV-2, B.1.1.298	BEI Resources	NR-53953
SARS-CoV-2, B.1.1.7 (alpha)	BEI Resources	NR-54000
SARS-CoV-2, B.1.351 (beta)	BEI Resources	NR-54008
SARS-CoV-2, P.1 (gamma)	BEI Resources	NR-54982
SARS-CoV-2, B.1.617.2 (delta)	BEI Resources	NR-55611
HKU5-SARS1-S	BEI Resources	NR-48814
MERS-CoV	BEI Resources	NR-48813
Influenza A virus/WSN/1933 (IAV)	A. Boon (Wustl)	N/A
icSARS-CoV-2 mNG	World Reference Center f	N/A
SARS-CoV-2 E802D	Gandhi, S. et al, 2022	N/A

Supplementary Table 2

CRISRP guide RNA sequence

lentiCRISPRv2 sgRNA	Target sequence	Source
ARID1A sgRNA #1	CAGCAGAACTCTCACGACCA	Yale Keck Oligo
ARID1A sgRNA #2	TTTGAATGCAAGATTGAACG	Yale Keck Oligo
ARID2 sgRNA #1	ACATAATGCATATATAACAC	Yale Keck Oligo
ARID2 sgRNA #2	CTACAGCATGCAATTCAATG	Yale Keck Oligo
BRD9 sgRNA #1	GGGAGGTAATGAATTGTCTG	Yale Keck Oligo
BRD9 sgRNA #2	GCAGACAGAGGTGTCTACAC	Yale Keck Oligo
HNF1A sgRNA #1	AGCAGCACAACATCCCACAG	Yale Keck Oligo
HNF1A sgRNA #2	GCGGACGTACCAGGTGTACA	Yale Keck Oligo
HNF1B sgRNA #1	AGCAACACAACATCCCCCAG	Yale Keck Oligo
HNF1B sgRNA #2	GCCGCAACCGGTTCAAATGG	Yale Keck Oligo
SMARCA4 sgRNA #1	CTAGGTATGAAGTAGCTCCG	Yale Keck Oligo
SMARCA4 sgRNA #2	ACCCCCATCCAGAAGCCGCG	Yale Keck Oligo

RNP gRNA	Target sequence	Source
SMARCA4 AGM #1	AAGAAGCTCGAATTTACCCG	IDT
SMARCA4 AGM #2	GGGCGTACGAGTTTGACAAG	IDT

Supplementary Table 3

Oligos for qPCR		Sequence	Source
SARS-CoV-2 N1	Forward	5′-GACCCCAAAATCAGCGAAAT-3′	Yale Keck Oligo
	Reverse	5′-TCTGGTTACTGCCAGTTGAATCTG-3′	Yale Keck Oligo
	Probe	5′-6FAM-ACCCCGCATTACGTTTGGTGGACC-BHQ1-	Yale Keck Oligo
IVA PA	Forward	5′-GGCCGACTACACTCTCGATGA-3′	Yale Keck Oligo
	Reverse	5′-TGTCTTATGGTGAATAGCCTGGTTT-3′	Yale Keck Oligo
ACE2	Forward	5′-GGGATCAGAGATCGGAAGAAGA-3′	Yale Keck Oligo
	Reverse	5′-AAGGAGGTCTGAACATCATCAGTG-3′	Yale Keck Oligo
DPP4	Forward	5′-GAATTATCCGGTCGAGTTTT-3′	Yale Keck Oligo
	Reverse	5′-GCCATCCTTTTAAAGAAGAG-3′	Yale Keck Oligo
HNF1A	Forward	5′-AGACGCTAGTGGAGGAGTGCAA-3′	Yale Keck Oligo
	Reverse	5′-GGCAAACCAGTTGTAGACACGC-3′	Yale Keck Oligo
SLC4A4	Forward	5′- GGAAAGCCAAGTCCTACCACGA-3′	Yale Keck Oligo
	Reverse	5′- TACCAGCAATCAGGTCGTGCCT-3′	Yale Keck Oligo
ACTIN	Forward	5′-GAGCACAGAGCCTCGCCTTT-3′	Yale Keck Oligo
	Reverse	5′-ATCATCATCCATGGTGAGCTGG-3′	Yale Keck Oligo
Mouse Actin	Forward	5′- ACTGTCGAGTCGCGTCCA -3′	Yale Keck Oligo
	Reverse	5′- ATCCATGGCGAACTGGTGG-3′	Yale Keck Oligo
Mouse Ace2	Forward	5′-ACCTTCGCAGAGATCAAGCC-3′	Yale Keck Oligo
	Reverse	5′-CCAGTGGGGCTGATGTAGGA-3′	Yale Keck Oligo

Supplementary Table 4

Antibody	Vendor	Catalog numb	Application
Anti-ACE2	ProSci	Cat#3217	Western blot: 1:2000
Anti-GAPDH antibody	BioLegend	Cat#607902	Western blot: 1:1000
Anti-LMNB1 antibody	BioLegend	Cat#869801	Western blot: 1:1000
Anti-Flag antibody	Sigma-Aldrich	Cat# F3165	Western blot: 1:1000
Anti-SMARCA4 antibody	Santa Cruz Biotechnolog	Cat#sc-17796	Western blot: 1:2000; Co-IP: 1:200
Anti-SMARCA4 antibody	Cell Signaling Technolog	Cat#49360	Western blot: 1:2000; CUT&Tag: 4uL/sample
Anti-SMARCC1 antibody	Cell Signaling Technolog	Cat#11956	Western blot: 1:2000; CUT&Tag: 4uL/sample
Anti-ARID1A antibody	Cell Signaling Technolog	Cat#12354	Western blot: 1:2000; CUT&Tag: 4uL/sample
Anti-H3K27ac antibody	Cell Signaling Technolog	Cat#8173	CUT&Tag: 4uL/sample
Anti-H3K4me1 antibody	Abcam	Cat#ab17687	CUT&Tag: 0.5 ug/sample
Anti-HNF1A antibody	Abcam	Cat#ab27269	CUT&Tag: 0.5 ug/sample
Guinea Pig anti-Rabbit IgG	Antibodies-online	Cat#ABIN101	CUT&Tag: 0.5 ug/sample
Anti-V5 antibody	Cell Signaling Technolog	Cat#13202	Western blot: 1:1000; Co-IP:1:200
Anti-GAPDH	Santa Cruz Biotechnolog	Cat#sc365062	Western blot: 1:5000
Anti-HNF1A antibody	Cell Signaling Technolog	Cat# 89670	Western blot: 1:1000
Anti-HNF1B antibody	Thermo Fisher Scientific	Cat# 720259	Western blot: 1:1000
Anti-HA antibody	BioLegend	Cat# 901513	Western blot: 1:1000
Goat anti-mouse IgG antibody (IRD	LI-COR Biosciences	Cat#926-6807	Western blot 1:10000
Goat anti-rabbit IgG antibody (IRD	LI-COR Biosciences	Cat#926-3221	Western blot 1:10000
Goat anti-rabbit IgG/HRP	Jackson ImmunoResearc	Cat#111-035-	Western blot 1:10000
Goat anti-rat IgG/HRP	Jackson ImmunoResearc	Cat#112-035-	Western blot 1:10000
Goat anti-mouse IgG/HRP	Jackson ImmunoResearc	Cat#115-035-	Western blot 1:10000

Supplementary Table 5

Figure	Comparison	Significance Stars	P-Value
1b	Control vs. ACE2-sgRNA	****	<0.0001
	Control vs. SMARCA4-sg#1	****	<0.0001
	Control vs. SMARCA4-sg#2	****	<0.0001
	Control vs. ARID1A-sg#1	****	<0.0001
	Control vs. ARID1A-sg#2	****	<0.0001
	Control vs. ARID2-sg#1	**	0.0027
	Control vs. ARID2-sg#2	**	0.0056
	Control vs. BRD9-sg#1	ns	0.4343
	Control vs. BRD9-sg#2	**	0.0055
1d	WT vs. KO+Empty 24h	***	0.0003
	WT vs. KO+SMARCA4 WT 24h	ns	0.0725
	WT vs. KO+SMARCA4 K785R 24h	***	0.0003
	WT vs. KO+Empty 48h	***	0.0002
	WT vs. KO+SMARCA4 WT 48h	ns	0.6007
	WT vs. KO+SMARCA4 K785R 48h	***	0.0002
1f	WT vs. KO+Empty	***	0.0002
	WT vs. KO+SMARCA4 WT	ns	0.441
	KO+Empty vs. KO+SMARCA4 WT	***	0.0004
	KO+Empty vs. KO+SMARCA4 K785R	ns	0.6028
	KO+SMARCA4 WT vs. KO+SMARCA4 K785R	***	0.0002
	WT vs. KO+Empty	***	0.0003
	WT vs. KO+SMARCA4 WT	ns	0.153
	KO+Empty vs. KO+SMARCA4 WT	***	0.00017
	KO+Empty vs. KO+SMARCA4 K785R	ns	0.9038
	KO+SMARCA4 WT vs. KO+SMARCA4 K785R	**	0.001
	WT vs. KO+Empty	ns	0.8488
	WT vs. KO+SMARCA4 WT	ns	0.3252
	KO+Empty vs. KO+SMARCA4 WT	ns	0.7148
	KO+Empty vs. KO+SMARCA4 K785R	ns	0.7848
	KO+SMARCA4 WT vs. KO+SMARCA4 K785R	ns	0.2786
1g	WT vs. KO+Empty	****	<0.0001
	WT vs. KO+SMARCA4 WT	ns	0.8389
	WT vs. KO+SMARCA4 K785R	****	<0.0001
	KO+Empty vs. KO+SMARCA4 WT	****	<0.0001
	KO+Empty vs. KO+SMARCA4 K785R	ns	0.2862
	KO+SMARCA4 WT vs. KO+SMARCA4 K785R	****	<0.0001
	WT vs. KO+Empty	****	<0.0001
	WT vs. KO+SMARCA4 WT	ns	0.0711
	WT vs. KO+SMARCA4 K785R	****	<0.0001
	KO+Empty vs. KO+SMARCA4 WT	***	0.0001

	KO+Empty vs. KO+SMARCA4 K785R	ns	>0.9999
	KO+SMARCA4 WT vs. KO+SMARCA4 K785R	****	<0.0001
	WT vs. KO+Empty	ns	0.4915
	WT vs. KO+SMARCA4 WT	ns	0.9991
	WT vs. KO+SMARCA4 K785R	ns	0.9991
	KO+Empty vs. KO+SMARCA4 WT	ns	0.4239
	KO+Empty vs. KO+SMARCA4 K785R	ns	0.4239
	KO+SMARCA4 WT vs. KO+SMARCA4 K785R	ns	>0.9999
2j	WT vs. SMARCA4-KO	***	0.0005
	WT vs. SMARCA4-KO+ACE2	ns	0.7775
	SMARCA4-KO vs. SMARCA4-KO+ACE2	***	0.0004
	WT vs. SMARCA4-KO	***	0.00017
	WT vs. SMARCA4-KO+ACE2	ns	0.1721
	SMARCA4-KO vs. SMARCA4-KO+ACE2	***	0.00022
3d	Ctrl vs. HNF1A-KO#1	****	<0.0001
	Ctrl vs. HNF1A-KO#2	****	<0.0001
	Ctrl vs. HNF1B-KO#1	****	<0.0001
	Ctrl vs. HNF1B-KO#2	****	<0.0001
3e	Ctrl vs. HNF1A-KO#1	****	<0.0001
	Ctrl vs. HNF1A-KO#2	****	<0.0001
	Ctrl vs. HNF1B-KO#1	****	<0.0001
	Ctrl vs. HNF1B-KO#2	****	<0.0001
3f	Ctrl vs. HNF1A-KO	***	0.0008
	Ctrl vs. HNF1B-KO	***	0.0008
	Ctrl vs. HNF1A-KO	****	<0.0001
	Ctrl vs. HNF1B-KO	****	<0.0001
	Ctrl vs. HNF1A-KO#1	***	0.0003
	Ctrl vs. HNF1A-KO#2	***	0.0002
	Ctrl vs. HNF1B-KO#1	***	0.0006
	Ctrl vs. HNF1B-KO#2	***	0.0007
4b	0 vs. 4	****	<0.0001
	0 vs. 8	****	<0.0001
	0 vs. 12	****	<0.0001
	0 vs. 16	****	<0.0001
	0 vs. 20	****	<0.0001
	0 vs. 24	****	<0.0001
	0 vs. 48	****	<0.0001

4c	0 vs. 0.16	****	<0.0001
	0 vs. 0.31	****	<0.0001
	0 vs. 0.62	****	<0.0001
	0 vs. 1.25	****	<0.0001
	0 vs. 2.5	****	<0.0001
4d	DMSO vs. 1.25 uM Comp12	***	0.0005
	DMSO vs. 2.5 uM Comp12	****	<0.0001
	IgG vs. anti-ACE2	****	<0.0001
4e	DMSO vs. 1.25 uM Comp12	***	0.00019
	DMSO vs. 2.5 uM Comp12	***	0.00018
4f	DMSO vs. 1.25 uM Comp12	****	<0.0001
	DMSO vs. 2.5 uM Comp12	****	<0.0001
	DMSO vs. 1.25 uM Comp12	***	0.0004
	DMSO vs. 2.5 uM Comp12	***	0.0003
	DMSO vs. 1.25 uM Comp12	***	0.0002
	DMSO vs. 2.5 uM Comp12	****	<0.0001
4g	DMSO vs. 1.25 uM Comp14	****	<0.0001
	DMSO vs. 2.5 uM Comp14	****	<0.0001
	DMSO vs. 1.25 uM ABCI1	**	0.0018
	DMSO vs. 2.5 uM ABCI1	***	0.0001
	DMSO vs. 1.25 uM dBRD9	ns	0.1175
	DMSO vs. 2.5 uM dBRD9	ns	0.9638
	DMSO vs. 1.25 uM Comp14	***	0.0008
	DMSO vs. 2.5 uM Comp14	****	<0.0001
	DMSO vs. 1.25 uM ABCI1	****	<0.0001
	DMSO vs. 2.5 uM ABCI1	****	<0.0001
	DMSO vs. 1.25 uM dBRD9	ns	0.3881
	DMSO vs. 2.5 uM dBRD9	ns	0.0841
4h	DMSO vs. Comp14	***	0.0004
	DMSO vs. ABCI1	**	0.0015
	DMSO vs. dBRD9	ns	0.5225
	DMSO vs. Comp14	***	0.0004
	DMSO vs. ABCI1	***	0.0005
	DMSO vs. dBRD9	ns	0.6947
4i	WA01	****	<0.0001
	B	****	<0.0001
	B.1.5	****	<0.0001
	B.1.1.222	****	<0.0001
	B.1.1.298	****	<0.0001

	B.1.1.7 (Alpha)	****	<0.0001
	B.1.351 (Beta)	****	<0.0001
	P.1 (Gamma)	****	<0.0001
	B.1.617.2 (Delta)	****	<0.0001
	B.1.1.529 (Omicron)	****	<0.0001
4j	WA1-DMSO vs. WA1-Remdesivir	***	0.0001
	WA1-DMSO vs. WA1-Comp12	***	0.0001
	E802D-DMSO vs. E802D-Remdesivir	**	0.0099
	E802D-DMSO vs. E802D-Comp12	**	0.0092
	WA1-Remdesivir vs. E802D-Remdesivir	**	0.0065
	WA1-Remdesivir vs. E802D-Comp12	ns	0.9991
	E802D-Remdesivir vs. E802D-Comp12	**	0.0068
	WA1-DMSO vs. WA1-Remdesivir	***	0.00056
	WA1-DMSO vs. WA1-Comp12	***	0.00059
	E802D-DMSO vs. E802D-Remdesivir	**	0.0077
	E802D-DMSO vs. E802D-Comp12	***	0.0001
	WA1-Remdesivir vs. E802D-Remdesivir	**	0.0058
	WA1-Remdesivir vs. E802D-Comp12	ns	>0.9999
	E802D-Remdesivir vs. E802D-Comp12	**	0.0014
5b	DMSO vs. 2.5uM Comp12	****	<0.0001
	DMSO vs. 2.5uM Comp12	****	<0.0001
	DMSO vs. 2.5uM Comp12	***	0.0008
	DMSO vs. 2.5uM Comp12	***	0.0008
	DMSO vs. 2.5uM Comp12	***	0.0005
	DMSO vs. 2.5uM Comp12	***	0.0005
	DMSO vs. 2.5uM Comp12	****	<0.0001
	DMSO vs. 2.5uM Comp12	****	<0.0001
	DMSO vs. 2.5uM Comp12	****	<0.0001
	DMSO vs. 2.5uM Comp12	****	<0.0001
	DMSO vs. 2.5uM Comp12	****	<0.0001
	DMSO vs. 2.5uM Comp12	****	<0.0001
5c	DMSO vs. 2.5uM Comp12	***	0.0001
	DMSO vs. 2.5uM Comp12	***	0.0001
5f	DMSO vs. Remdesivir	***	0.0004
	DMSO vs. Comp12	***	0.0004
	DMSO vs. E802D-DMSO	*	0.0312
	DMSO vs. E802D-Remdesivir	***	0.0004
	DMSO vs. E802D-Comp12	***	0.0004
	Remdesivir vs. E802D-Remdesivir	**	0.0076
	E802D-DMSO vs. E802D-Remdesivir	**	0.0076
	E802D-DMSO vs. E802D-Comp12	**	0.0066
	E802D-Remdesivir vs. E802D-Comp12	**	0.0036

5g	DMSO vs. Comp12	****	<0.0001
	DMSO vs. Comp14	****	<0.0001
	DMSO vs. ABCI1	****	<0.0001
	DMSO vs. dBRD9	ns	0.9945
	DMSO vs. Comp12	****	<0.0001
	DMSO vs. Comp14	****	<0.0001
	DMSO vs. ABCI1	****	<0.0001
	DMSO vs. dBRD9	ns	0.9496
5i	DMSO vs. Comp12	****	<0.0001
	DMSO vs. Comp14	****	<0.0001
	DMSO vs. ABCI1	****	<0.0001
	DMSO vs. dBRD9	ns	0.3062
	DMSO vs. Comp12	***	0.0005
	DMSO vs. Comp14	***	0.0005
	DMSO vs. ABCI1	***	0.0009
	DMSO vs. dBRD9	ns	0.6631
	DMSO vs. Remdesivir	***	0.0005
5j	DMSO vs. Comp12	ns	0.5989
	DMSO vs. Comp14	ns	0.6039
	DMSO vs. ABCI1	ns	0.2535
	DMSO vs. dBRD9	ns	0.3528
	DMSO vs. Comp12	ns	0.9968
	DMSO vs. Comp14	ns	0.9997
	DMSO vs. ABCI1	ns	0.8811
	DMSO vs. dBRD9	ns	0.9968
	DMSO vs. Remdesivir	***	0.001
Extended Fig1c	Control vs. ACE2-sgRNA	****	<0.0001
	Control vs. SMARCA4-sg#1	****	<0.0001
	Control vs. SMARCA4-sg#2	****	<0.0001
	Control vs. ARID1A-sg#1	****	<0.0001
	Control vs. ARID1A-sg#2	****	<0.0001
	Control vs. ARID2-sg#1	ns	0.9994
	Control vs. ARID2-sg#2	ns	0.7409
	Control vs. BRD9-sg#1	ns	0.9845
	Control vs. BRD9-sg#2	ns	0.9032
	Control vs. ACE2-sgRNA	****	<0.0001
	Control vs. SMARCA4-sg#1	****	<0.0001
	Control vs. SMARCA4-sg#2	****	<0.0001
	Control vs. ARID1A-sg#1	***	0.0005
	Control vs. ARID1A-sg#2	***	0.0005
	Control vs. ARID2-sg#1	ns	0.9593

	Control vs. ARID2-sg#2	ns	>0.9999
	Control vs. BRD9-sg#1	ns	0.5718
	Control vs. BRD9-sg#2	ns	0.169
Extended Fig2a	Control vs. SMARCA4-sg#1	****	<0.0001
	Control vs. SMARCA4-sg#2	****	<0.0001
	Control vs. ARID2-sg#1	ns	0.9997
	Control vs. ARID2-sg#2	ns	0.5537
	Control vs. BRD9-sg#1	ns	0.3189
	Control vs. BRD9-sg#2	ns	0.9982
	Control vs. SMARCA4-sg#1	****	<0.0001
	Control vs. SMARCA4-sg#2	****	<0.0001
	Control vs. ARID2-sg#1	ns	0.7805
	Control vs. ARID2-sg#2	*	0.0398
	Control vs. BRD9-sg#1	ns	0.7805
	Control vs. BRD9-sg#2	*	0.0225
	Control vs. SMARCA4-sg#1	ns	0.4548
	Control vs. SMARCA4-sg#2	ns	0.8288
	Control vs. ARID2-sg#1	ns	>0.9999
	Control vs. ARID2-sg#2	ns	0.3476
	Control vs. BRD9-sg#1	ns	0.8288
	Control vs. BRD9-sg#2	ns	0.5777
Extended Fig2b	Control vs. SMARCA4-sg#1	***	0.0004
	Control vs. SMARCA4-sg#2	****	<0.0001
	Control vs. ARID2-sg#1	ns	0.1921
	Control vs. ARID2-sg#2	ns	0.9878
	Control vs. BRD9-sg#1	ns	0.4561
	Control vs. BRD9-sg#2	ns	0.2754
Extended Fig2c	Control vs. SMARCA4-sg#1	*	0.01
	Control vs. SMARCA4-sg#2	**	0.006
Extended Fig2d	Control vs. SMARCA4-sg#1	****	<0.0001
	Control vs. SMARCA4-sg#2	****	<0.0001
	Control vs. ACE2-sgRNA	****	<0.0001
	Control vs. CTSL-sgRNA	****	<0.0001
Extended Fig2f	Control vs. SMARCA4-sg#1	***	0.0006
	Control vs. SMARCA4-sg#2	***	0.0002
Extended Fig2g	Control vs. SMARCA4-sg#1	*	0.0396
	Control vs. SMARCA4-sg#2	**	0.0012
Extended Fig3b	WT vs. KO#1	****	<0.0001
	WT vs. KO#2	****	<0.0001
	WT vs. KO#3	****	<0.0001

	WT vs. KO#4	****	<0.0001
Extended Fig3c	WT vs. KO#1	****	<0.0001
	WT vs. KO#2	****	<0.0001
	WT vs. KO#3	****	<0.0001
	WT vs. KO#4	****	<0.0001
Extended Fig3d	WT vs. KO#1	****	<0.0001
	WT vs. KO#2	****	<0.0001
	WT vs. KO#3	****	<0.0001
	WT vs. KO#4	****	<0.0001
Extended Fig 5c	Ctrl vs. HNF1A-KO#1	***	0.0001
	Ctrl vs. HNF1A-KO#2	****	<0.0001
	Ctrl vs. HNF1B-KO#1	**	0.0019
	Ctrl vs. HNF1B-KO#2	***	0.0005
Extended Fig 5c	Ctrl vs. HNF1A-KO#1	**	0.0051
	Ctrl vs. HNF1A-KO#2	***	0.0007
	Ctrl vs. HNF1B-KO#1	**	0.0012
	Ctrl vs. HNF1B-KO#2	***	0.0004
Extended Fig 5e	Ctrl vs. HNF1A-KO#1	****	<0.0001
	Ctrl vs. HNF1A-KO#2	****	<0.0001
	Ctrl vs. HNF1B-KO#1	****	<0.0001
	Ctrl vs. HNF1B-KO#2	****	<0.0001
Extended Fig 6a	0 vs. 4	ns	0.9999
	0 vs. 8	ns	>0.9999
	0 vs. 12	ns	0.9998
	0 vs. 16	ns	0.9794
	0 vs. 20	ns	0.7438
	0 vs. 24	ns	0.8725
	0 vs. 48	ns	0.0661
Extended Fig 6b	0 vs. 0.16	ns	0.2066
	0 vs. 0.31	ns	0.9485
	0 vs. 0.62	ns	0.9982
	0 vs. 1.25	ns	0.9961
	0 vs. 2.5	ns	0.7262
Extended Fig 6b	DMSO vs. Comp14	*	0.0107
	DMSO vs. ABCI1	*	0.0144
	DMSO vs. dBRD9	ns	0.9001
	DMSO vs. Comp14	****	<0.0001
	DMSO vs. ABCI1	****	<0.0001
	DMSO vs. dBRD9	ns	0.6937

Extended Fig 6e	DMSO vs. Comp14	***	0.0001
	DMSO vs. ABCI1	***	0.0002
	DMSO vs. dBRD9	ns	0.7815
	DMSO vs. Comp14	****	<0.0001
	DMSO vs. ABCI1	****	<0.0001
	DMSO vs. dBRD9	*	0.0455
Extended Fig 8b	DMSO vs. 1	****	<0.0001
	DMSO vs. 2	****	<0.0001
	DMSO vs. 3	****	<0.0001
	DMSO vs. 4	****	<0.0001
	DMSO vs. 1	***	0.0006
	DMSO vs. 2	***	0.0001
	DMSO vs. 3	****	<0.0001
	DMSO vs. 4	****	<0.0001
Extended Fig 8c	DMSO vs. 1	****	<0.0001
	DMSO vs. 2	****	<0.0001
	DMSO vs. 3	****	<0.0001
	DMSO vs. 4	****	<0.0001
	DMSO vs. 1	****	<0.0001
	DMSO vs. 2	****	<0.0001
	DMSO vs. 3	****	<0.0001
	DMSO vs. 4	****	<0.0001
Extended Fig 8f	DMSO vs. Comp12	**	0.0082
	DMSO vs. Comp14	**	0.0068
	DMSO vs. ABCI1	**	0.0086
	DMSO vs. dBRD9	ns	>0.9999
	DMSO vs. Comp12	****	<0.0001
	DMSO vs. Comp14	****	<0.0001
	DMSO vs. ABCI1	****	<0.0001
	DMSO vs. dBRD9	ns	>0.9999
Extended Fig 9c	DMSO vs. Comp14 (-4 dpi)	ns	0.8767
	DMSO vs. Comp14 (-1 dpi)	ns	0.994
	DMSO vs. Comp14 (0 dpi)	ns	0.6106
Extended Fig 9c	DMSO vs. Comp14 (-4 dpi)	ns	0.85
	DMSO vs. Comp14 (-1 dpi)	ns	0.9971
	DMSO vs. Comp14 (0 dpi)	ns	0.753
Extended Fig 9e	Lung	ns	0.2703
	Liver	**	0.0047
	Heart	ns	0.8601

SI	ns	0.2083
