# Supplementary material for: Pharmacological disruption of mSWI/SNF complex activity restricts SARS-CoV-2 infection
Source: Nat Genet. 2023 Mar 9;55(3):471–83. doi: 10.1038/s41588-023-01307-z (PMC10011139; doi:10.1038/s41588-023-01307-z)
Supplement: Supplementary file 2 — Reporting Summary [file 41588_2023_1307_MOESM2_ESM.pdf]

nature portfolio

Corresponding author(s): Craig Wilen

Last updated by author(s): Oct 14, 2022

Reporting Summary

Nature Portfolio wishes to improve the reproducibility of the work that we publish. This form provides structure for consistency and transparency in reporting. For further information on Nature Portfolio policies, see our Editorial Policies and the Editorial Policy Checklist.

Statistics

For all statistical analyses, confirm that the following items are present in the figure legend, table legend, main text, or Methods section.

n/a	Confirmed	
☐	☒	The exact sample size (*n*) for each experimental group/condition, given as a discrete number and unit of measurement
☐	☒	A statement on whether measurements were taken from distinct samples or whether the same sample was measured repeatedly
☐	☒	The statistical test(s) used AND whether they are one- or two-sided<br>*Only common tests should be described solely by name; describe more complex techniques in the Methods section.*
☒	☐	A description of all covariates tested
☒	☐	A description of any assumptions or corrections, such as tests of normality and adjustment for multiple comparisons
☐	☒	A full description of the statistical parameters including central tendency (e.g. means) or other basic estimates (e.g. regression coefficient) AND variation (e.g. standard deviation) or associated estimates of uncertainty (e.g. confidence intervals)
☐	☒	For null hypothesis testing, the test statistic (e.g. *F*, *t*, *r*) with confidence intervals, effect sizes, degrees of freedom and *P* value noted<br>*Give P values as exact values whenever suitable.*
☒	☐	For Bayesian analysis, information on the choice of priors and Markov chain Monte Carlo settings
☒	☐	For hierarchical and complex designs, identification of the appropriate level for tests and full reporting of outcomes
☒	☐	Estimates of effect sizes (e.g. Cohen's *d*, Pearson's *r*), indicating how they were calculated

Our web collection on statistics for biologists contains articles on many of the points above.

Software and code

Policy information about availability of computer code

Data collection	All packages used for data analysis and collection are listed below in ChIP-seq section.

Data analysis

NGS Data Processing
CUT&Tag, ATAC-Seq and human RNA-Seq samples were sequenced with the Illumina NextSeq 500 technology, and RNA-seq samples from Vero E6 cells were sequenced with Illumina NovaSeq 6000 technology. For the RNA-Seq data, reads were aligned to either the chlSab2 (NCBI annotation release 100) or the hg19 reference genome using STAR aligner v2.7.3a with parameters --winAnchorMultimapNmax 200 --outFilterMultimapNmax 100 --quantMode GeneCounts, and bigWig files were generated using the deeptools v3.1.3 bamCoverage function with the normalizeUsingRPKM option. Output gene count tables from STAR were used as input into the edgeR v3.12.1 R software package to evaluate differential gene expression. For ATAC-Seq data, read trimming was carried out by Trimmomatic v0.36, followed by alignment, duplicate read removal, and read quality filtering using Bowtie2, Picard v2.8.0 (http://broadinstitute.github.io/picard/), and SAMtools v 0.1.19, respectively, and ATAC-seq peaks were called with MACS2 v2.1 using the BAMPE option and a broad peak cutoff of 0.001. For ATAC-Seq track generation, output BAM files were converted into BigWig files using MACS2 and UCSC utilities in order to display coverage throughout the genome in RPM values. For CUT&Tag libraries, the CutRunTools pipeline was leveraged to perform read trimming, quality filtering, alignment, peak calling, and track building using default parameters.

CUT&Tag and ATAC-seq Data Analyses
Heatmaps and metaplots displaying signals aligned to peak centers were generated using ngsplot v2.63. K-means clustering was applied to output RPM values in order to partition the data into groups. The Bedtools multiIntersectBed and merge functions were used for peak merging, and distance-to-TSS peak distributions were computed utilizing Ensembl gene coordinates provided by the UCSC genome browser. Principle Component Analysis was performed using the wt.scale and fast.svd functions from the corpcor R package on log2-transformed RPKM values within merged peaks. Transcription factor motif positions within peaks were identified by the MEME FIMO v 4.12.0 software tool with position frequency matrices curated previously, and motif fractions of occurrence within clusters of peaks were computed using in-house scripts.

For manuscripts utilizing custom algorithms or software that are central to the research but not yet described in published literature, software must be made available to editors and reviewers. We strongly encourage code deposition in a community repository (e.g. GitHub). See the Nature Portfolio guidelines for submitting code & software for further information.

Data

Policy information about availability of data

All manuscripts must include a data availability statement. This statement should provide the following information, where applicable:

- Accession codes, unique identifiers, or web links for publicly available datasets
- A description of any restrictions on data availability
- For clinical datasets or third party data, please ensure that the statement adheres to our policy

All genomics data has been deposited under Gene Expression Omnibus GSE186201.

Field-specific reporting

Please select the one below that is the best fit for your research. If you are not sure, read the appropriate sections before making your selection.

☒ Life sciences ☐ Behavioural & social sciences ☐ Ecological, evolutionary & environmental sciences

For a reference copy of the document with all sections, see nature.com/documents/nr-reporting-summary-flat.pdf

Life sciences study design

All studies must disclose on these points even when the disclosure is negative.

Sample size

No sample-size calculations were performed, sample sizes were chosen based on the norm in the field of experimentation. All biochemical studies are performed in technical and experimental at least n>=3 replicates and genomic studies (ATAC,RNA) were carried out in duplicate across the cell lines and across multiple BAF subunits (one replicate each) for CUT&Tag studies, most appropriate for the evaluation of the biologic inquiries at hand.

Data exclusions

No data exclusions were conducted in this study.

Replication

All biochemical studies we were performed in triplicate (n=3) or greater for all experiments. Genomic studies from cell lines were all carried out in duplicate (ATAC, RNA) or probing mulitple BAF complex members independently (one replicate each) (CUT&Tag). All attempts at replication for data shown in the study were successful. Extensive measures were taken to verify reproducibility of results, including the utility of mutliple cell lines, where appropriate, multiple experiments performed by different individuals, and consistent validation of constructs used.

Randomization

Mice were randomized based on sex in the in vivo experiments, randomization was not required at any other stage of this study. Control of covariates was also not required since experiments performed were designed to measure the impact of genetic perturbations and small molecules across experimental conditions in cell lines or primary cells and not primary specimens or human patients.

Blinding

Blinding was not necessary in this study since it was not a clinical trial involving human patients. Investigators needed to have knowledge of the experimental groups and conditions.

Reporting for specific materials, systems and methods

We require information from authors about some types of materials, experimental systems and methods used in many studies. Here, indicate whether each material, system or method listed is relevant to your study. If you are not sure if a list item applies to your research, read the appropriate section before selecting a response.

Materials & experimental systems

n/a	Involved in the study
☐	☒ Antibodies
☐	☒ Eukaryotic cell lines
☒	☐ Palaeontology and archaeology
☐	☒ Animals and other organisms
☒	☐ Human research participants
☒	☐ Clinical data
☒	☐ Dual use research of concern

Methods

n/a	Involved in the study
☐	☒ ChIP-seq
☒	☐ Flow cytometry
☒	☐ MRI-based neuroimaging

Antibodies

Antibodies used

Antibody; Vendor; Catalog number; Application.
Anti-ACE2, ProSci, Cat#3217, Western blot: 1:2000
Anti-GAPDH antibody, BioLegend, Cat#607902, Western blot: 1:1000
Anti-LMNB1 antibody, BioLegend, Cat#869801, Western blot: 1:1000
Anti-Flag antibody, Sigma-Aldrich, Cat# F3165, Western blot: 1:1000
Anti-SMARCA4 antibody, Santa Cruz Biotechnology, Cat#sc-17796, Western blot: 1:2000, Co-IP: 1:200
Anti-SMARCA4 antibody, Cell Signaling Technology, Cat#49360, Western blot: 1:2000, CUT&Tag: 4uL/sample
Anti-SMARCC1 antibody, Cell Signaling Technology, Cat#11956, Western blot: 1:2000, CUT&Tag: 4uL/sample
Anti-ARID1A antibody, Cell Signaling Technology, Cat#12354, Western blot: 1:2000, CUT&Tag: 4uL/sample
Anti-H3K27ac antibody, Cell Signaling Technology, Cat#8173, CUT&Tag: 4uL/sample
Anti-H3K4me1 antibody, Abcam, Cat#ab176877, CUT&Tag: 0.5 ug/sample
Anti-HNF1A antibody, Abcam, Cat#ab272693, CUT&Tag: 0.5 ug/sample
Guinea Pig anti-Rabbit IgG, Antibodies-online, Cat#ABIN101961, CUT&Tag: 0.5 ug/sample
Anti-V5 antibody, Cell Signaling Technology, Cat#13202, Western blot: 1:1000, Co-IP:1:200
Anti-GAPDH, Santa Cruz Biotechnology, Cat#sc365062, Western blot: 1:5000
Anti-HNF1A antibody, Cell Signaling Technology, Cat# 89670, Western blot: 1:1000
Anti-HNF1B antibody, Thermo Fisher Scientific, Cat# 720259, Western blot: 1:1000
Anti-HA antibody, BioLegend, Cat# 901513, Western blot: 1:1000
Goat anti-mouse IgG antibody, IRDye 680RD conjugated, LI-COR Biosciences, Cat#926-68070, Western blot 1:10,000
Goat anti-rabbit IgG antibody, IRDye 800RD conjugated, LI-COR Biosciences, Cat#926-32211, Western blot 1:10,000
Goat anti-rabbit IgG/HRP, Jackson ImmunoResearch, Cat#111-035-003, Western blot 1:10,000
Goat anti-rat IgG/HRP, Jackson ImmunoResearch, Cat#112-035-003, Western blot 1:10,000
Goat anti-mouse IgG/HRP, Jackson ImmunoResearch, Cat#115-035-003, Western blot 1:10,000

Validation

All primary antibodies were validated in our laboratory for on-target specificity using cell lines in which the target was inactivated. These studies were performed in addition to the validation studies performed by the commercial vendor (use catalog numbers listed above to access this data on the websites of the vendors).

Eukaryotic cell lines

Policy information about cell lines

Cell line source(s)

HEK293T (ATCC, CRL-3216)
Vero E6 (ATCC, CRL-1586)
Huh7.5 (Washington University in St. Louis cell culture facility)
Calu-3 (ATCC, HTB-55)
HBEC (Lonza CC-2541)
H9 (WiCell)
Human Intestinal Enteroid (J2) (Texas Medical Center Digestive Diseases Center Enteroid Core).

Authentication

All cell lines were subjected to standard cell line fingerprinting analyses via our in-house cell line identify verification pipeline (Yale University and Dana-Farber Molecular Diagnostics Laboratory). ATCC performed STR profiling for HEK293T and Calu-3 cells. No Vero E6 or HBECs authentication was found on the vendors websites.

Mycoplasma contamination

All cell lines used in this study were routinely tested for mycoplasma contamination and were confirmed to be negative throughout the study.

Commonly misidentified lines
(See ICLAC register)

None of the cell lines used in this study are known to be commonly misidentified.

Animals and other organisms

Policy information about studies involving animals; ARRIVE guidelines recommended for reporting animal research

Laboratory animals	8–10 week old C57BL/6J mice (purchased from Jackson labs) of both genders were used in vivo experiments. All infections experiments were conducted in animal biosafety level 3 (ABSL-3) facilities at Yale University. All mice were housed in individually ventilated microisolator cages in a facility maintained at Yale University School of Medicine, on a 12 h light/dark cycle maintained at 40-60% humidity and 72 degrees F+/-2 degrees. Regular chow diet and water were given until the defined experimental endpoints. Researchers were not blinded during in vivo experiments.
Wild animals	No wild animals were used in this study
Field-collected samples	No field collected samples were used in this study
Ethics oversight	All animal work was approved by Institutional Animal Care and Use Committee (IACUC) at Yale University School of Medicine according to guidelines. All infection work was performed in an animal biosafety level 3 (ABSL-3) facility at Yale University School of Medicine.

Note that full information on the approval of the study protocol must also be provided in the manuscript.

ChIP-seq

Data deposition

☒ Confirm that both raw and final processed data have been deposited in a public database such as GEO.

☒ Confirm that you have deposited or provided access to graph files (e.g. BED files) for the called peaks.

Data access links
May remain private before publication.

All genomics data has been deposited under GSE186201,
https://www.ncbi.nlm.nih.gov/geo/query/acc.cgi?acc=GSE186201

Files in database submission

GSM5640292 ATACseq.VERO.HNF1AKO.Rep1
GSM5640293 ATACseq.VERO.HNF1AKO.Rep2
GSM5640294 ATACseq.VERO.LentiCRISPRv2.Control.Rep1
GSM5640295 ATACseq.VERO.LentiCRISPRv2.Control.Rep2
GSM5640296 ATACseq.VERO.SMARCA4KO.EmptyVector.Rep1
GSM5640297 ATACseq.VERO.SMARCA4KO.EmptyVector.Rep2
GSM5640298 ATACseq.VERO.SMARCA4KO.SMARCA4K785R.Rep1
GSM5640299 ATACseq.VERO.SMARCA4KO.SMARCA4K785R.Rep2
GSM5640300 ATACseq.VERO.SMARCA4KO.SMARCA4WT.Rep1
GSM5640301 ATACseq.VERO.SMARCA4KO.SMARCA4WT.Rep2
GSM5640302 CutAndTag.VERO.HNF1AKO.H3K27ac
GSM5640303 CutAndTag.VERO.HNF1AKO.H3K4me1
GSM5640304 CutAndTag.VERO.HNF1AKO.SMARCA4
GSM5640305 CutAndTag.VERO.HNF1AKO.SMARCC1
GSM5640306 CutAndTag.VERO.LentiCRISPRv2.Control.H3K27ac
GSM5640307 CutAndTag.VERO.LentiCRISPRv2.Control.H3K4me1
GSM5640308 CutAndTag.VERO.LentiCRISPRv2.Control.SMARCA4
GSM5640309 CutAndTag.VERO.LentiCRISPRv2.Control.SMARCC1
GSM5640310 CutAndTag.VERO.SMARCA4KO.EmptyVector.ARID1A
GSM5640311 CutAndTag.VERO.SMARCA4KO.EmptyVector.H3K27ac
GSM5640312 CutAndTag.VERO.SMARCA4KO.EmptyVector.H3K4me1
GSM5640313 CutAndTag.VERO.SMARCA4KO.EmptyVector.SMARCA4
GSM5640314 CutAndTag.VERO.SMARCA4KO.EmptyVector.SMARCC1
GSM5640315 CutAndTag.VERO.SMARCA4KO.SMARCA4K785R.ARID1A
GSM5640316 CutAndTag.VERO.SMARCA4KO.SMARCA4K785R.H3K27ac
GSM5640317 CutAndTag.VERO.SMARCA4KO.SMARCA4K785R.H3K4me1
GSM5640318 CutAndTag.VERO.SMARCA4KO.SMARCA4K785R.SMARCA4
GSM5640319 CutAndTag.VERO.SMARCA4KO.SMARCA4K785R.SMARCC1
GSM5640320 CutAndTag.VERO.SMARCA4KO.SMARCA4WT.ARID1A
GSM5640321 CutAndTag.VERO.SMARCA4KO.SMARCA4WT.H3K27ac
GSM5640322 CutAndTag.VERO.SMARCA4KO.SMARCA4WT.H3K4me1
GSM5640323 CutAndTag.VERO.SMARCA4KO.SMARCA4WT.SMARCA4
GSM5640324 CutAndTag.VERO.SMARCA4KO.SMARCA4WT.SMARCC1
GSM5640325 RNAseq.VERO.SMARCA4KO.EmptyVector.Rep1
GSM5640326 RNAseq.VERO.SMARCA4KO.EmptyVector.Rep2
GSM5640327 RNAseq.VERO.SMARCA4KO.SMARCA4K785R.Rep1
GSM5640328 RNAseq.VERO.SMARCA4KO.SMARCA4K785R.Rep2
GSM5640329 RNAseq.VERO.SMARCA4KO.SMARCA4WT.Rep1
GSM5640330 RNAseq.VERO.SMARCA4KO.SMARCA4WT.Rep2
GSM5640331 RNAseq.VERO.WT.Rep1
GSM5640332 RNAseq.VERO.WT.Rep2
GSM6254270 ATACseq.CALU3.C12.Rep1
GSM6254271 ATACseq.CALU3.C12.Rep2

GSM6254272 ATACseq.CALU3.DMSO.Rep1
GSM6254273 ATACseq.CALU3.DMSO.Rep2
GSM6254274 ATACseq.HUH7.C12.Rep1
GSM6254275 ATACseq.HUH7.C12.Rep2
GSM6254276 ATACseq.HUH7.DMSO.Rep1
GSM6254277 ATACseq.HUH7.DMSO.Rep2
GSM6254278 CutAndTag.CALU3.C12.ARID1A
GSM6254279 CutAndTag.CALU3.C12.H3K27ac
GSM6254280 CutAndTag.CALU3.C12.HNF1A
GSM6254281 CutAndTag.CALU3.C12.IgG
GSM6254282 CutAndTag.CALU3.C12.SMARCA4
GSM6254283 CutAndTag.CALU3.C12.SMARCC1
GSM6254284 CutAndTag.CALU3.DMSO.ARID1A
GSM6254285 CutAndTag.CALU3.DMSO.H3K27ac
GSM6254286 CutAndTag.CALU3.DMSO.HNF1A
GSM6254287 CutAndTag.CALU3.DMSO.IgG
GSM6254288 CutAndTag.CALU3.DMSO.SMARCA4
GSM6254289 CutAndTag.CALU3.DMSO.SMARCC1
GSM6254290 CutAndTag.HUH7.C12.ARID1A
GSM6254291 CutAndTag.HUH7.C12.H3K27ac
GSM6254292 CutAndTag.HUH7.C12.HNF1A
GSM6254293 CutAndTag.HUH7.C12.IgG
GSM6254294 CutAndTag.HUH7.C12.SMARCA4
GSM6254295 CutAndTag.HUH7.C12.SMARCC1
GSM6254296 CutAndTag.HUH7.DMSO.ARID1A
GSM6254297 CutAndTag.HUH7.DMSO.H3K27ac
GSM6254298 CutAndTag.HUH7.DMSO.HNF1A
GSM6254299 CutAndTag.HUH7.DMSO.IgG
GSM6254300 CutAndTag.HUH7.DMSO.SMARCA4
GSM6254301 CutAndTag.HUH7.DMSO.SMARCC1
GSM6254302 RNAseq.CALU3.C12.Rep1
GSM6254303 RNAseq.CALU3.C12.Rep2
GSM6254304 RNAseq.CALU3.DMSO.Rep1
GSM6254305 RNAseq.CALU3.DMSO.Rep2
GSM6254306 RNAseq.HBEC.C12.Rep1
GSM6254307 RNAseq.HBEC.C12.Rep2
GSM6254308 RNAseq.HBEC.DMSO.Rep1
GSM6254309 RNAseq.HBEC.DMSO.Rep2
GSM6254310 RNAseq.HUH7.C12.Rep1
GSM6254311 RNAseq.HUH7.C12.Rep2
GSM6254312 RNAseq.HUH7.DMSO.Rep1
GSM6254313 RNAseq.HUH7.DMSO.Rep2

Genome browser session
(e.g. UCSC)

N/A

Methodology

Replicates

All ATAC-Seq and RNA-seq experiments were performed with at least two experimental replicates.

Sequencing depth

Sample ;Raw; Mapped; Uniquely Mapped; Length; Type
ATACseq.VERO.SMARCA4KO.EmptyVector.Rep1 50064446 47960635 18811100 35 Paired-End
ATACseq.VERO.SMARCA4KO.EmptyVector.Rep2 54553022 52199365 21231096 35 Paired-End
ATACseq.VERO.SMARCA4KO.SMARCA4WT.Rep1 97178490 93511756 21931011 35 Paired-End
ATACseq.VERO.SMARCA4KO.SMARCA4WT.Rep2 48942350 47275025 12850909 35 Paired-End
ATACseq.VERO.SMARCA4KO.SMARCA4K785R.Rep1 46208267 44452426 12905870 35 Paired-End
ATACseq.VERO.SMARCA4KO.SMARCA4K785R.Rep2 53378858 51234025 16266283 35 Paired-End
ATACseq.VERO.LentiCRISPRv2.Control.Rep1 40703413 39303072 27152478 35 Paired-End
ATACseq.VERO.LentiCRISPRv2.Control.Rep2 46296458 44684410 32434560 35 Paired-End
ATACseq.VERO.HNF1AKO.Rep1 50040529 48183631 34580401 35 Paired-End
ATACseq.VERO.HNF1AKO.Rep2 35637193 34419439 25112053 35 Paired-End
CutAndTag.VERO.LentiCRISPRv2.Control.SMARCA4 15843744 15151271 3598525 35 Paired-End
CutAndTag.VERO.LentiCRISPRv2.Control.SMARCC1 1641509 1577751 987386 35 Paired-End
CutAndTag.VERO.LentiCRISPRv2.Control.H3K27ac 21821593 21181148 16267911 35 Paired-End
CutAndTag.VERO.LentiCRISPRv2.Control.H3K4me1 36782896 35501871 29345293 35 Paired-End
CutAndTag.VERO.HNF1AKO.SMARCA4 7600038 7262921 2276367 35 Paired-End
CutAndTag.VERO.HNF1AKO.SMARCC1 11897081 11406285 5600223 35 Paired-End
CutAndTag.VERO.HNF1AKO.H3K27ac 25502037 24727475 18616363 35 Paired-End
CutAndTag.VERO.HNF1AKO.H3K4me1 37867845 36540432 30818736 35 Paired-End
CutAndTag.VERO.SMARCA4KO.EmptyVector.SMARCA4 83033 74900 54129 35 Paired-End
CutAndTag.VERO.SMARCA4KO.SMARCA4WT.SMARCA4 8470011 8009993 2821037 35 Paired-End
CutAndTag.VERO.SMARCA4KO.SMARCA4K785R.SMARCA4 711296 668318 334248 35 Paired-End
CutAndTag.VERO.SMARCA4KO.EmptyVector.SMARCC1 8924313 8378840 5050556 35 Paired-End
CutAndTag.VERO.SMARCA4KO.EmptyVector.ARID1A 10483042 9948455 2529762 35 Paired-End
CutAndTag.VERO.SMARCA4KO.EmptyVector.H3K27ac 8760810 8440780 7779031 35 Paired-End
CutAndTag.VERO.SMARCA4KO.EmptyVector.H3K4me1 9679958 9418710 8480066 35 Paired-End

CutAndTag.VERO.SMARCA4KO.SMARCA4WT.SMARCC1 10070988 9476553 3780608 35 Paired-End
CutAndTag.VERO.SMARCA4KO.SMARCA4WT.ARID1A 10177954 9478853 1929227 35 Paired-End
CutAndTag.VERO.SMARCA4KO.SMARCA4WT.H3K27ac 10034560 9651293 8358126 35 Paired-End
CutAndTag.VERO.SMARCA4KO.SMARCA4WT.H3K4me1 11494931 10863752 9330520 35 Paired-End
CutAndTag.VERO.SMARCA4KO.SMARCA4K785R.SMARCC1 9763351 9214464 6155927 35 Paired-End
CutAndTag.VERO.SMARCA4KO.SMARCA4K785R.ARID1A 12028716 11281821 2983073 35 Paired-End
CutAndTag.VERO.SMARCA4KO.SMARCA4K785R.H3K27ac 10135395 9716169 8669656 35 Paired-End
CutAndTag.VERO.SMARCA4KO.SMARCA4K785R.H3K4me1 11735226 11321496 10337708 35 Paired-End
RNAseq.VERO.SMARCA4KO.EmptyVector.Rep1 34972676 32117051 30588498 195 Paired-End
RNAseq.VERO.SMARCA4KO.EmptyVector.Rep2 39372910 37047505 34781556 195 Paired-End
RNAseq.VERO.SMARCA4KO.SMARCA4K785R.Rep1 41272795 38920061 37201048 195 Paired-End
RNAseq.VERO.SMARCA4KO.SMARCA4K785R.Rep2 35911806 34089197 32522672 195 Paired-End
RNAseq.VERO.SMARCA4KO.SMARCA4WT.Rep1 29688599 27850389 26549323 195 Paired-End
RNAseq.VERO.SMARCA4KO.SMARCA4WT.Rep2 37186405 35158173 33428929 195 Paired-End
RNAseq.VERO.WT.Rep1 35001460 33276812 31937466 195 Paired-End
RNAseq.VERO.WT.Rep2 30525617 28792825 27513620 195 Paired-End
ATACseq.HUH7.DMSO.Rep1 73552084 72910492 39745383 35 Paired-End
ATACseq.HUH7.DMSO.Rep2 94977254 94074492 50054494 35 Paired-End
ATACseq.HUH7.C12.Rep1 114859074 113578633 56233500 35 Paired-End
ATACseq.HUH7.C12.Rep2 71218896 70661392 33309222 35 Paired-End
ATACseq.CALU3.DMSO.Rep1 104751662 103861624 82231751 35 Paired-End
ATACseq.CALU3.DMSO.Rep2 95012850 94174177 76705963 35 Paired-End
ATACseq.CALU3.C12.Rep1 86556466 85804953 70609291 35 Paired-End
ATACseq.CALU3.C12.Rep2 108666628 107456305 89666621 35 Paired-End
CutAndTag.HUH7.DMSO.SMARCA4 5085029 4918511 2425331 35 Paired-End
CutAndTag.HUH7.DMSO.ARID1A 5663126 5461988 2475931 35 Paired-End
CutAndTag.HUH7.DMSO.SMARCC1 2241316 2169597 1248939 35 Paired-End
CutAndTag.HUH7.DMSO.HNF1A 3532572 3400988 1300143 35 Paired-End
CutAndTag.HUH7.DMSO.H3K27ac 25896526 25562504 20723209 35 Paired-End
CutAndTag.HUH7.DMSO.IgG 26795 25815 17162 35 Paired-End
CutAndTag.HUH7.C12.SMARCA4 5325436 5113629 2462887 35 Paired-End
CutAndTag.HUH7.C12.ARID1A 6005413 5783054 3432016 35 Paired-End
CutAndTag.HUH7.C12.SMARCC1 8836719 8527034 3725423 35 Paired-End
CutAndTag.HUH7.C12.HNF1A 12455961 12037067 5292795 35 Paired-End
CutAndTag.HUH7.C12.H3K27ac 17183864 16936723 12582524 35 Paired-End
CutAndTag.HUH7.C12.IgG 31809 30732 20404 35 Paired-End
CutAndTag.CALU3.DMSO.SMARCA4 7296925 7117743 2772901 35 Paired-End
CutAndTag.CALU3.DMSO.ARID1A 4461567 4357105 1900877 35 Paired-End
CutAndTag.CALU3.DMSO.SMARCC1 931569 907737 636903 35 Paired-End
CutAndTag.CALU3.DMSO.HNF1A 4671727 4479874 1838186 35 Paired-End
CutAndTag.CALU3.DMSO.H3K27ac 22349971 22105999 15002181 35 Paired-End
CutAndTag.CALU3.DMSO.IgG 7712 7494 6493 35 Paired-End
CutAndTag.CALU3.C12.SMARCA4 5154448 4980944 1547165 35 Paired-End
CutAndTag.CALU3.C12.ARID1A 2547564 2443563 1005072 35 Paired-End
CutAndTag.CALU3.C12.SMARCC1 844705 817911 466813 35 Paired-End
CutAndTag.CALU3.C12.HNF1A 3007548 2911133 1528035 35 Paired-End
CutAndTag.CALU3.C12.H3K27ac 17481621 17242408 11051405 35 Paired-End
CutAndTag.CALU3.C12.IgG 9045 7855 6349 35 Paired-End
RNAseq.HUH7.DMSO.Rep1 27473853 27281475 21718543 75 Single-End
RNAseq.HUH7.DMSO.Rep2 30866627 30665354 24449432 75 Single-End
RNAseq.HUH7.C12.Rep1 27881121 27685709 21158144 75 Single-End
RNAseq.HUH7.C12.Rep2 31563767 31340468 23870488 75 Single-End
RNAseq.CALU3.DMSO.Rep1 32724097 32471665 24937325 75 Single-End
RNAseq.CALU3.DMSO.Rep2 33967040 33707698 25763308 75 Single-End
RNAseq.CALU3.C12.Rep1 30218878 29987337 22481939 75 Single-End
RNAseq.CALU3.C12.Rep2 30671807 30430405 22859537 75 Single-End
RNAseq.HBEC.DMSO.Rep1 37562554 37276351 28924591 75 Single-End
RNAseq.HBEC.DMSO.Rep2 32775690 32525618 25390844 75 Single-End
RNAseq.HBEC.C12.Rep1 32092433 31869140 24173958 75 Single-End
RNAseq.HBEC.C12.Rep2 38469728 38223369 29057408 75 Single-End

Antibodies

Antibody for CUT&Tag; Vendor; Catalog number; Lot number
anti-SMARCA4 (Cell Signaling Technology Catalog#: 41360 Lot:3),
anti-ARID1A (Cell Signaling Technology Catalog#: 12354 Lot:3),
anti-SMARCC1 (Cell Signaling Technology Catalog#: 11956 Lot:4),
anti-H3K27ac (Cell Signaling Technology Catalog#: 8173 Lot:6),
anti-H3K4me1 (Abcam Catalog#: ab176877 Lot:GR208955-5)

Peak calling parameters

For ATAC-Seq data, read trimming was carried out by Trimmomatic v0.36, followed by alignment, duplicate read removal, and read quality filtering using Bowtie2, Picard v2.8.0, and SAMtools v 0.1.19, respectively. ATAC-seq peaks were called with MACS2 v2.1 using the BAMPE option and a broad peak cutoff of 0.001. For CUT&Tag libraries, the CutRunTools pipeline was leveraged to perform read trimming, quality filtering, alignment, duplicate read removal, peak calling, and track building using default parameters. From this pipeline, we used narrow peaks called by MACS2 with a q-value cutoff of 0.01. For the RNA-Seq data, reads were aligned to either the chlSab2 (NCBI annotation release 100) or the hg19 reference genome using STAR aligner v2.7.3a with parameters --winAnchorMultimapNmax 200 --outFilterMultimapNmax 100 --quantMode GeneCounts, and bigWig files were generated using the

deeptools v3.1.3 bamCoverage function with the normalizeUsingRPKM option. Output gene count tables from STAR were used as input into the edgeR v3.12.1 R software package to evaluate differential gene expression.

Data quality

Sample	Peak.Count
ATACseq.VERO.SMARCA4KO.EmptyVector.Rep1	21276
ATACseq.VERO.SMARCA4KO.EmptyVector.Rep2	22174
ATACseq.VERO.SMARCA4KO.SMARCA4WT.Rep1	42491
ATACseq.VERO.SMARCA4KO.SMARCA4WT.Rep2	32296
ATACseq.VERO.SMARCA4KO.SMARCA4K785R.Rep1	21693
ATACseq.VERO.SMARCA4KO.SMARCA4K785R.Rep2	23110
ATACseq.VERO.LentiCRISPRv2.Control.Rep1	63567
ATACseq.VERO.LentiCRISPRv2.Control.Rep2	71290
ATACseq.VERO.HNF1AKO.Rep1	70060
ATACseq.VERO.HNF1AKO.Rep2	58042
CutAndTag.VERO.LentiCRISPRv2.Control.SMARCA4	23243
CutAndTag.VERO.LentiCRISPRv2.Control.SMARCC1	11860
CutAndTag.VERO.LentiCRISPRv2.Control.H3K27ac	70129
CutAndTag.VERO.LentiCRISPRv2.Control.H3K4me1	165824
CutAndTag.VERO.HNF1AKO.SMARCA4	22895
CutAndTag.VERO.HNF1AKO.SMARCC1	28224
CutAndTag.VERO.HNF1AKO.H3K27ac	67782
CutAndTag.VERO.HNF1AKO.H3K4me1	173560
CutAndTag.VERO.SMARCA4KO.EmptyVector.SMARCA4	119
CutAndTag.VERO.SMARCA4KO.SMARCA4WT.SMARCA4	25905
CutAndTag.VERO.SMARCA4KO.SMARCA4K785R.SMARCA4	4049
CutAndTag.VERO.SMARCA4KO.EmptyVector.SMARCC1	8072
CutAndTag.VERO.SMARCA4KO.EmptyVector.ARID1A	1502
CutAndTag.VERO.SMARCA4KO.EmptyVector.H3K27ac	35055
CutAndTag.VERO.SMARCA4KO.EmptyVector.H3K4me1	76005
CutAndTag.VERO.SMARCA4KO.SMARCA4WT.SMARCC1	16538
CutAndTag.VERO.SMARCA4KO.SMARCA4WT.ARID1A	6369
CutAndTag.VERO.SMARCA4KO.SMARCA4WT.H3K27ac	56642
CutAndTag.VERO.SMARCA4KO.SMARCA4WT.H3K4me1	53281
CutAndTag.VERO.SMARCA4KO.SMARCA4K785R.SMARCC1	13239
CutAndTag.VERO.SMARCA4KO.SMARCA4K785R.ARID1A	3662
CutAndTag.VERO.SMARCA4KO.SMARCA4K785R.H3K27ac	40032
CutAndTag.VERO.SMARCA4KO.SMARCA4K785R.H3K4me1	74073
ATACseq.CALU3.C12.Rep1.broadPeak	52932
ATACseq.CALU3.C12.Rep2.broadPeak	56324
ATACseq.CALU3.DMSO.Rep1.broadPeak	90676
ATACseq.CALU3.DMSO.Rep2.broadPeak	87435
ATACseq.HUH7.C12.Rep1.broadPeak	25955
ATACseq.HUH7.C12.Rep2.broadPeak	22498
ATACseq.HUH7.DMSO.Rep1.broadPeak	52593
ATACseq.HUH7.DMSO.Rep2.broadPeak	56964
CutAndTag.CALU3.C12.ARID1A.narrowPeak	3271
CutAndTag.CALU3.C12.H3K27ac.narrowPeak	34824
CutAndTag.CALU3.C12.HNF1A.narrowPeak	445
CutAndTag.CALU3.C12.IgG.narrowPeak	0
CutAndTag.CALU3.C12.SMARCA4.narrowPeak	7773
CutAndTag.CALU3.C12.SMARCC1.narrowPeak	301
CutAndTag.CALU3.DMSO.ARID1A.narrowPeak	14917
CutAndTag.CALU3.DMSO.H3K27ac.narrowPeak	53863
CutAndTag.CALU3.DMSO.HNF1A.narrowPeak	3287
CutAndTag.CALU3.DMSO.IgG.narrowPeak	0
CutAndTag.CALU3.DMSO.SMARCA4.narrowPeak	20035
CutAndTag.CALU3.DMSO.SMARCC1.narrowPeak	1444
CutAndTag.HUH7.C12.ARID1A.narrowPeak	1982
CutAndTag.HUH7.C12.H3K27ac.narrowPeak	45330
CutAndTag.HUH7.C12.HNF1A.narrowPeak	5088
CutAndTag.HUH7.C12.IgG.narrowPeak	1
CutAndTag.HUH7.C12.SMARCA4.narrowPeak	3272
CutAndTag.HUH7.C12.SMARCC1.narrowPeak	7484
CutAndTag.HUH7.DMSO.ARID1A.narrowPeak	10897
CutAndTag.HUH7.DMSO.H3K27ac.narrowPeak	76144
CutAndTag.HUH7.DMSO.HNF1A.narrowPeak	7662
CutAndTag.HUH7.DMSO.IgG.narrowPeak	2
CutAndTag.HUH7.DMSO.SMARCA4.narrowPeak	12134
CutAndTag.HUH7.DMSO.SMARCC1.narrowPeak	6136

Software

NGS Data Processing
CUT&Tag, ATAC-Seq and human RNA-Seq samples were sequenced with the Illumina NextSeq 500 technology, and RNA-seq samples from Vero E6 cells were sequenced with Illumina NovaSeq 6000 technology. For the RNA-Seq data, reads were aligned to either the chlSab2 (NCBI annotation release 100) or the hg19 reference genome using STAR aligner v2.7.3a with parameters --winAnchorMultimapNmax 200 --outFilterMultimapNmax 100 --quantMode GeneCounts, and bigWig files were generated using the deeptools v3.1.3 bamCoverage function with the normalizeUsingRPKM option. Output gene count tables from STAR were used as

input into the edgeR v3.12.1 R software package to evaluate differential gene expression. For ATAC-Seq data, read trimming was carried out by Trimmomatic v0.36, followed by alignment, duplicate read removal, and read quality filtering using Bowtie2, Picard v2.8.0 (http://broadinstitute.github.io/picard/), and SAMtools v 0.1.19, respectively, and ATAC-seq peaks were called with MACS2 v2.1 using the BAMPE option and a broad peak cutoff of 0.001. For ATAC-Seq track generation, output BAM files were converted into BigWig files using MACS2 and UCSC utilities in order to display coverage throughout the genome in RPM values. For Cut and Tag libraries, the CutRunTools pipeline was leveraged to perform read trimming, quality filtering, alignment, peak calling, and track building using default parameters.

CUT&Tag and ATAC-seq Data Analyses
Heatmaps and metaplots displaying signals aligned to peak centers were generated using ngsplot v2.63. K-means clustering was applied to output RPM values in order to partition the data into groups. The Bedtools multiIntersectBed and merge functions were used for peak merging, and distance-to-TSS peak distributions were computed utilizing Ensembl gene coordinates provided by the UCSC genome browser. Principle Component Analysis was performed using the wt.scale and fast.svd functions from the corpcor R package on log2-transformed RPKM values within merged peaks. Transcription factor motif positions within peaks were identified by the MEME FIMO v 4.12.0 software tool with position frequency matrices curated previously, and motif fractions of occurrence within clusters of peaks were computed using in-house scripts.
