# Supplementary figures and images for: Pharmacological disruption of mSWI/SNF complex activity restricts SARS-CoV-2 infection
Source: Nat Genet. 2023 Mar 9;55(3):471–83. doi: 10.1038/s41588-023-01307-z (PMC10011139; doi:10.1038/s41588-023-01307-z)

**Fig. 1c**

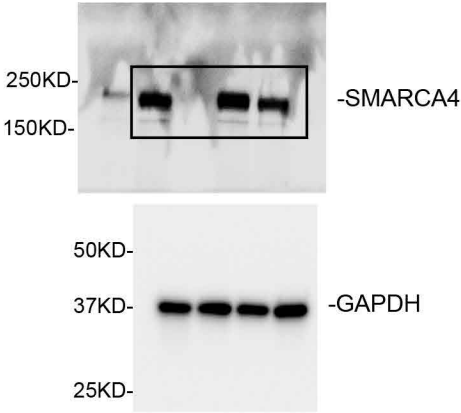

Supplement: Source Data Fig. 1 — Unprocessed western blots. [file 41588_2023_1307_MOESM16_ESM.pdf]

**Fig. 2a**

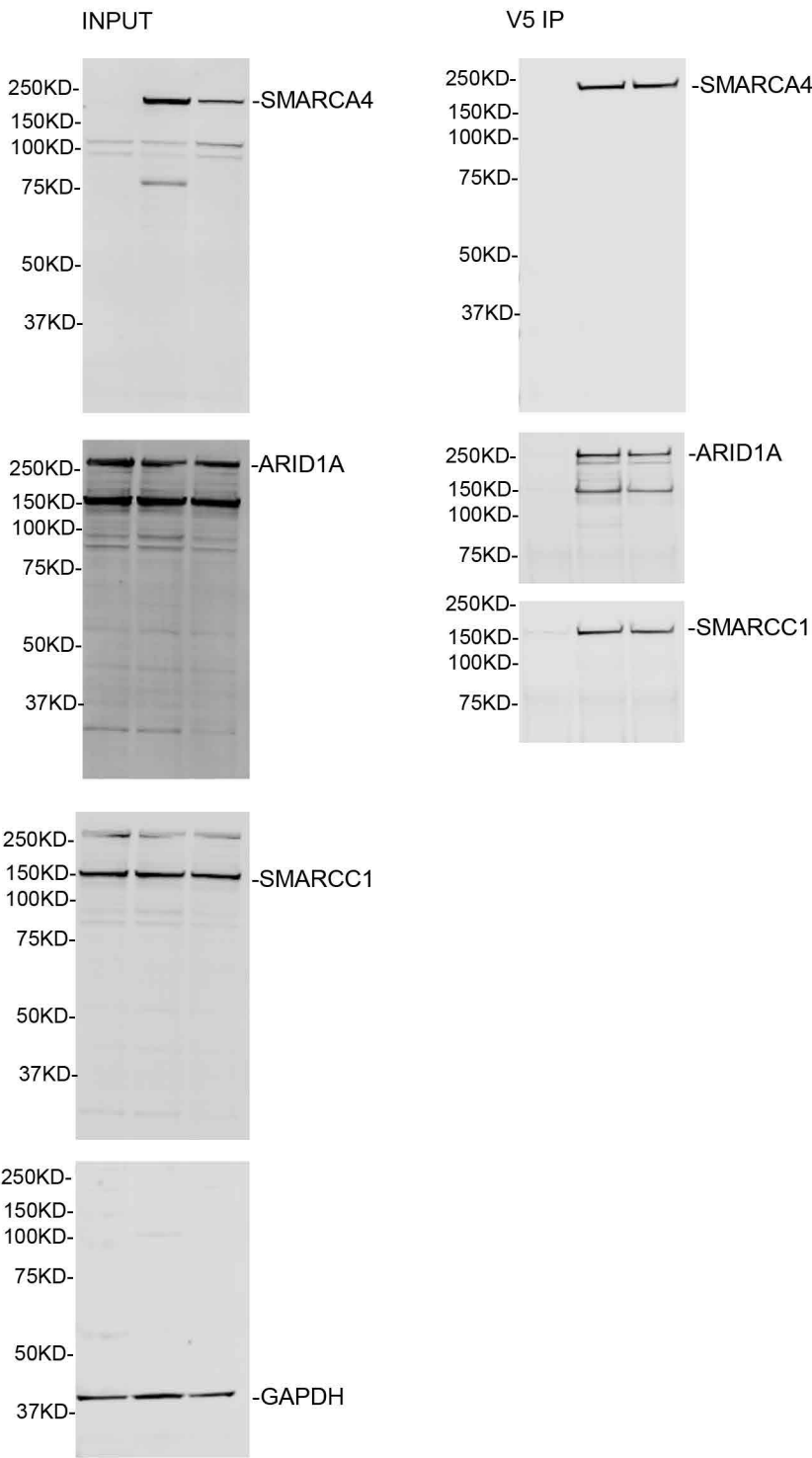

**Fig. 2i**

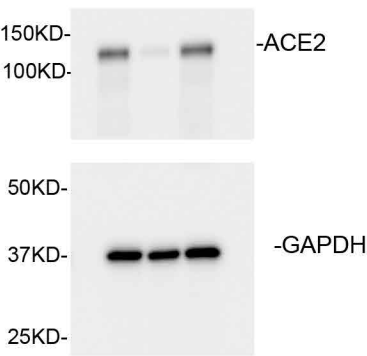

Supplement: Source Data Fig. 2 — Unprocessed western blots. [file 41588_2023_1307_MOESM17_ESM.pdf]

**Fig. 3c**

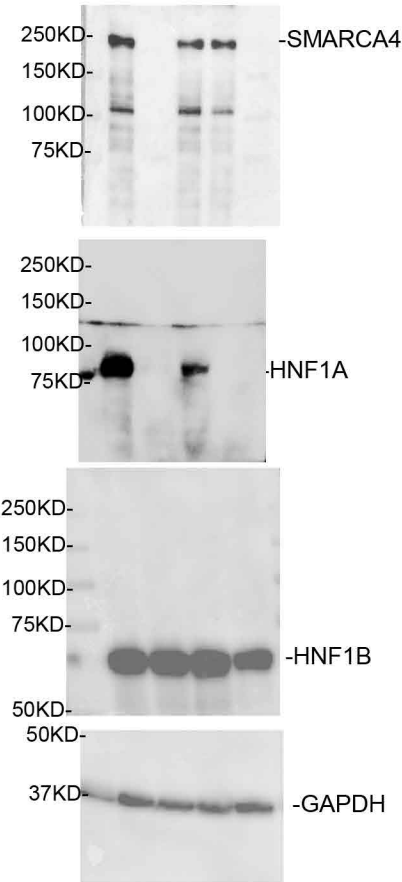

**Fig. 3d**

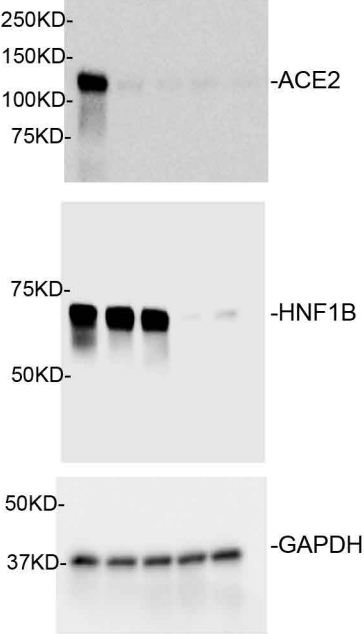

**Fig. 3h**

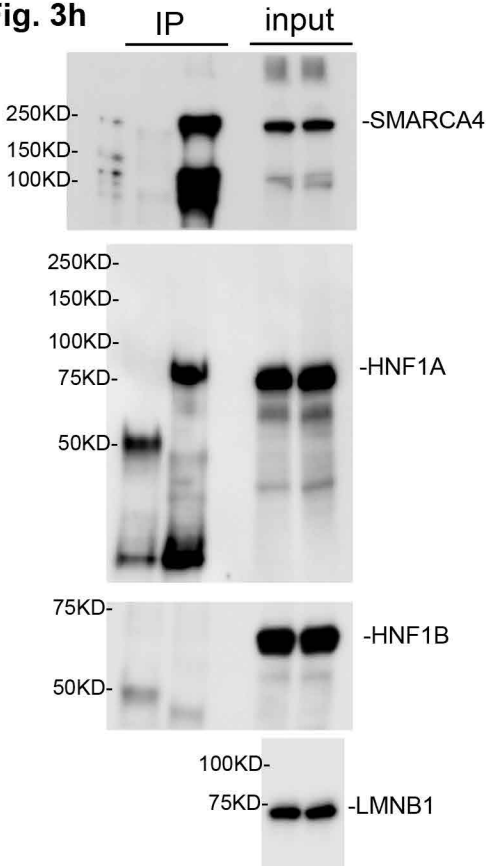

Supplement: Source Data Fig. 3 — Unprocessed western blots. [file 41588_2023_1307_MOESM18_ESM.pdf]

**Fig. 4b**

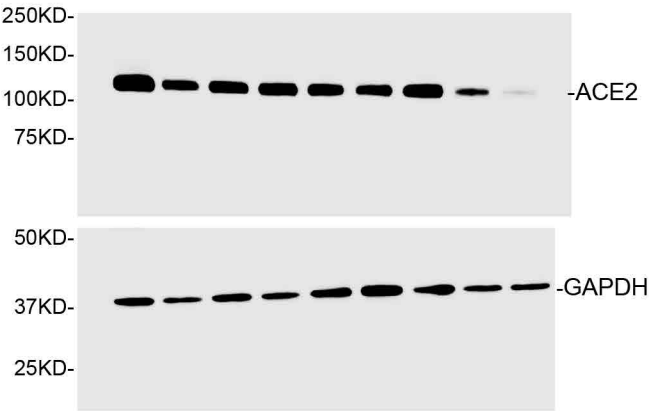

**Fig. 4f**

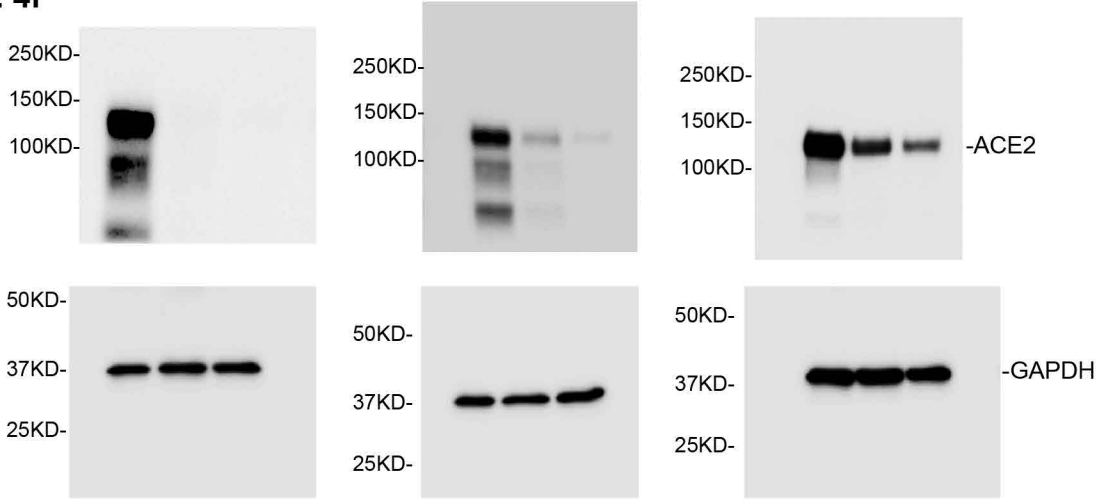

**Fig. 4g**

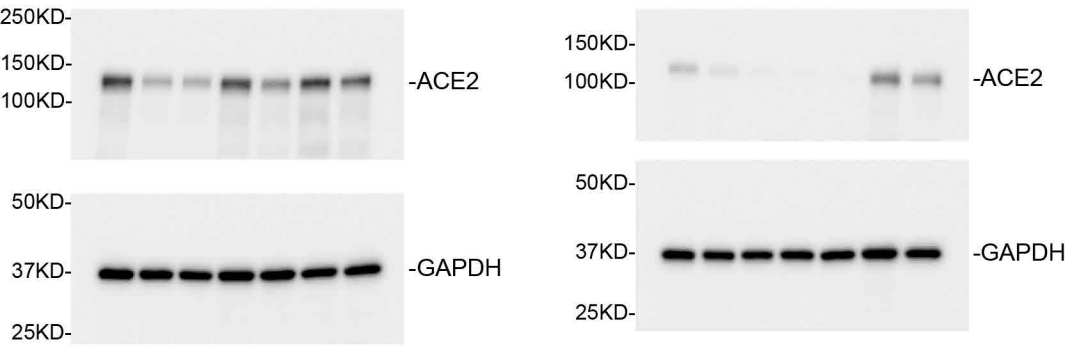

Supplement: Source Data Fig. 4 — Unprocessed western blots. [file 41588_2023_1307_MOESM19_ESM.pdf]

**Extended Fig. 1e**

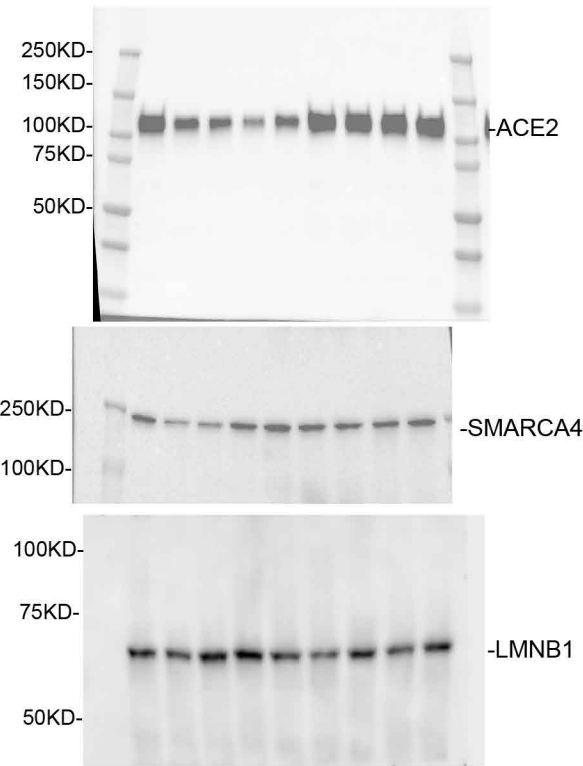

Supplement: Source Data Extended Data Fig. 1 — Unprocessed western blots. [file 41588_2023_1307_MOESM20_ESM.pdf]

**Extended Fig. 2e**

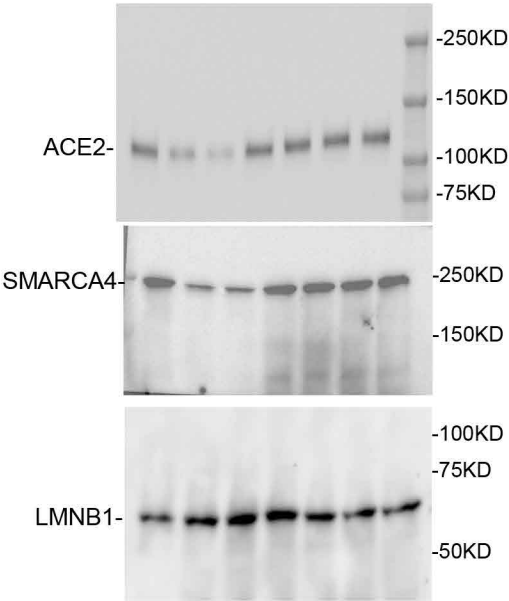

Supplement: Source Data Extended Data Fig. 2 — Unprocessed western blots. [file 41588_2023_1307_MOESM21_ESM.pdf]

**Extended Fig. 3a**

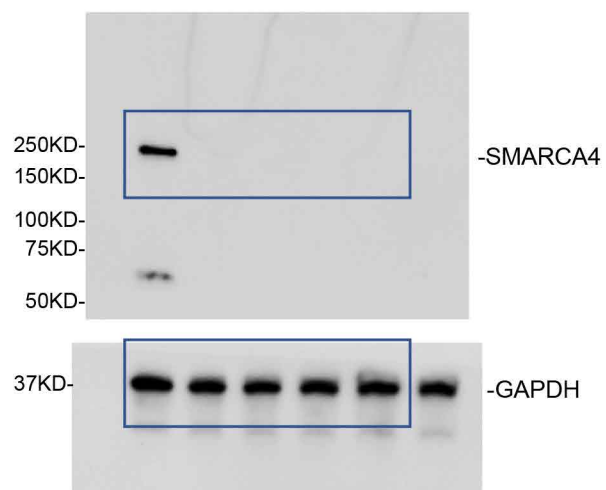

Supplement: Source Data Extended Data Fig. 3 — Unprocessed western blots. [file 41588_2023_1307_MOESM22_ESM.pdf]

**Extended Fig. 5b**

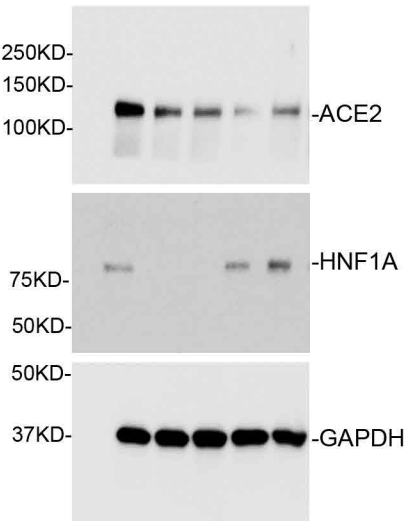

**Extended Fig. 5f**

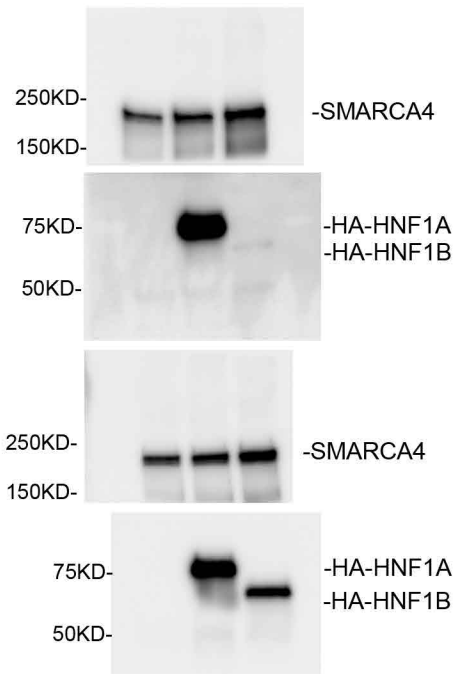

**Extended Fig. 5g**

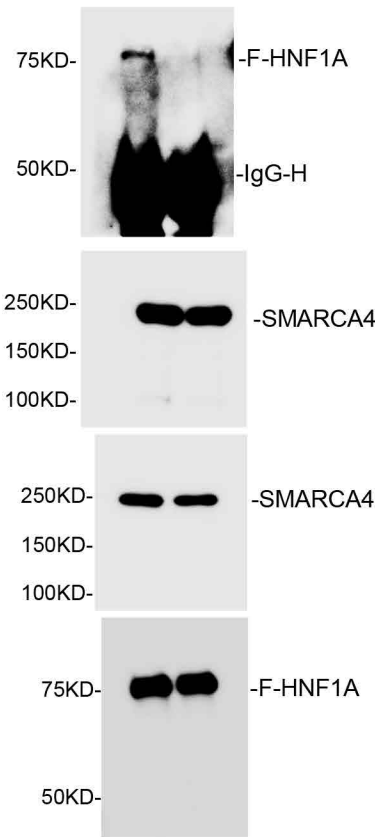

Supplement: Source Data Extended Data Fig. 5 — Unprocessed western blots. [file 41588_2023_1307_MOESM23_ESM.pdf]
